# UNC45A deficiency causes microvillus inclusion disease–like phenotype by impairing myosin VB–dependent apical trafficking

**DOI:** 10.1172/JCI154997

**Published:** 2022-05-16

**Authors:** Rémi Duclaux-Loras, Corinne Lebreton, Jérémy Berthelet, Fabienne Charbit-Henrion, Ophelie Nicolle, Céline Revenu des Courtils, Stephanie Waich, Taras Valovka, Anis Khiat, Marion Rabant, Caroline Racine, Ida Chiara Guerrera, Júlia Baptista, Maxime M. Mahe, Michael W. Hess, Béatrice Durel, Nathalie Lefort, Céline Banal, Mélanie Parisot, Cecile Talbotec, Florence Lacaille, Emmanuelle Ecochard-Dugelay, Arzu Meltem Demir, Georg F. Vogel, Laurence Faivre, Astor Rodrigues, Darren Fowler, Andreas R. Janecke, Thomas Müller, Lukas A. Huber, Fernando Rodrigues-Lima, Frank M. Ruemmele, Holm H. Uhlig, Filippo Del Bene, Grégoire Michaux, Nadine Cerf-Bensussan, Marianna Parlato

**Affiliations:** 1 Université Paris Cité, Imagine Institute, Laboratory of Intestinal Immunity, INSERM, UMR1163, Paris, France.; 2 Department of Pediatric Gastroenterology, Assistance Publique-Hopitaux de Paris, Hopital Necker–Enfants Malades, F-75015, Paris, France.; 3 Université Paris Cité, CEDC, UMR 7216, CNRS, Paris, France.; 4 Université de Rennes, CNRS, Institut de Génétique et Développement de Rennes (IGDR)–UMR 6290, Rennes, France.; 5 Sorbonne Université, INSERM, CNRS, Institut de la Vision, Paris, France.; 6 Institut Curie, PSL Research University, INSERM U934, CNRS UMR3215, Paris, France.; 7 Universitätsklinik für Pädiatrie I and; 8 Institute of Cell Biology, Biocenter, Medical University of Innsbruck, Innsbruck, Austria.; 9 Department of Pathology, Assistance Publique–Hopitaux de Paris, Hopital Necker–Enfants Malades, Paris, France.; 10 Centre de Référence Anomalies du Développement et Syndromes Malformatifs, Fédération Hospitalo–Universitaire Médecine Translationnelle et Anomalies du Développement (TRANSLAD), Centre Hospitalier Universitaire, and Equipe GAD, Université de Bourgogne Franche-Comté, Faculté de Médecine, INSERM LNC UMR 1231, Dijon, France.; 11 Proteomics Platform 3P5-Necker, Université Paris Descartes-Structure Fédérative de Recherche Necker, INSERM US24/CNRS UMS3633, Paris, France.; 12 Peninsula Medical School, Faculty of Health, University of Plymouth, Plymouth, United Kingdom.; 13 Royal Devon and Exeter NHS Foundation Trust, Exeter, United Kingdom.; 14 Université de Nantes, INSERM, TENS, The Enteric Nervous System in Gut and Brain Diseases, IMAD, Nantes, France.; 15 Institut für Histologie und Embryologie Medical University of Innsbruck, Innsbruck, Austria.; 16 Cell Imaging Platform, INSERM-US24-CNRS UMS 3633 Structure Fédérative de Recherche Necker, Université Paris Cité, Paris, France.; 17 iPS Core Facility, Imagine Institute, INSERM U1163, Paris Descartes University, Paris, France.; 18 Genomics Core Facility, Institut Imagine–Structure Fédérative de Recherche Necker, INSERM U1163 et INSERM US24/CNRS UMS3633, Paris Descartes Sorbonne Paris Cite University, Paris, France.; 19 Hopital Robert Debré, Assistance Publique Hôpitaux de Paris, Paris, France.; 20 Ankara Child Health and Diseases, Training and Research Hospital, Pediatric Gastroenterology, Ankara, Turkey.; 21 John Radcliffe Hospital, Oxford, United Kingdom.; 22 Université Paris Cité, BFA, UMR 8251, CNRS, Paris, France.; 23 Translational Gastroenterology Unit and Department of Paediatrics, John Radcliffe Hospital, NIHR Oxford Biomedical Research Centre, University of Oxford, Oxford, United Kingdom.

**Keywords:** Gastroenterology, Epithelial transport of ions and water

## Abstract

Variants in the UNC45A cochaperone have been recently associated with a syndrome combining diarrhea, cholestasis, deafness, and bone fragility. Yet the mechanism underlying intestinal failure in UNC45A deficiency remains unclear. Here, biallelic variants in *UNC45A* were identified by next-generation sequencing in 6 patients with congenital diarrhea. Corroborating in silico prediction, variants either abolished UNC45A expression or altered protein conformation. Myosin VB was identified by mass spectrometry as client of the UNC45A chaperone and was found misfolded in UNC45A^KO^ Caco-2 cells. In keeping with impaired myosin VB function, UNC45A^KO^ Caco-2 cells showed abnormal epithelial morphogenesis that was restored by full-length UNC45A, but not by mutant alleles. Patients and *UNC45A*^KO^ 3D organoids displayed altered luminal development and microvillus inclusions, while 2D cultures revealed Rab11 and apical transporter mislocalization as well as sparse and disorganized microvilli. All those features resembled the subcellular abnormalities observed in duodenal biopsies from patients with microvillus inclusion disease. Finally, microvillus inclusions and shortened microvilli were evidenced in enterocytes from *unc45a*-deficient zebrafish. Taken together, our results provide evidence that UNC45A plays an essential role in epithelial morphogenesis through its cochaperone function of myosin VB and that UNC45A loss causes a variant of microvillus inclusion disease.

## Graphical abstract

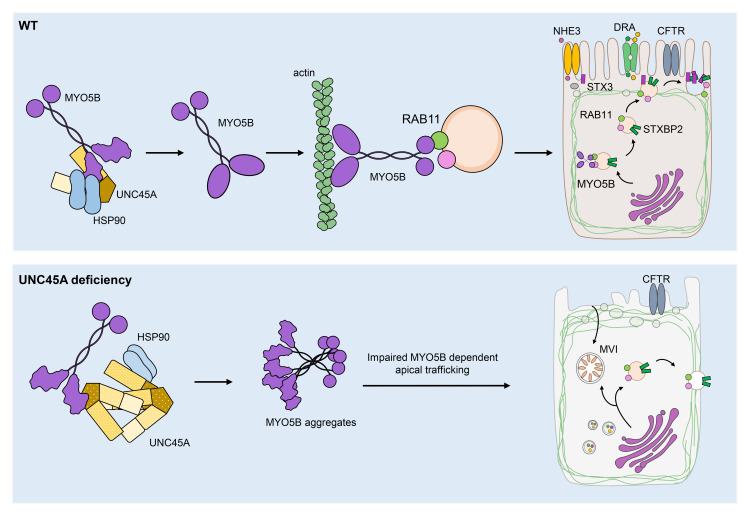

## Introduction

Congenital diarrhea disorders (CDDs) are rare monogenic diseases characterized by chronic, life-threatening diarrhea starting early in life (1, 2). Depending on the mechanism, diarrhea can be the only symptom or one manifestation of a more complex syndrome involving several organs. A syndrome that variably combines congenital diarrhea, cholestasis, loss of hearing, and bone fragility was recently described in 3 families and ascribed to loss-of-function (LoF) variants in *UNC45A* (3). A zebrafish model of unc45a deficiency showed abnormal development of epithelial folds in the proximal intestine and impaired intestinal motility (3). Yet the exact mechanism underlying intestinal symptoms remains poorly characterized. UNC45A belongs to the conserved UNC45/CRO1/She4p (UCS) protein family of myosin cochaperones, which participates in myosin-dependent functions, including cytokinesis, endocytosis, and muscle organization (4). All UNC45 proteins share the same 3-domain organization, with a C-terminal UCS domain, which binds the motor domain of myosin, a less-conserved central domain of unknown function and an N-terminal tetratricopeptide repeat (TPR) domain that binds to Hsp90. In concert with UNC45, Hsp90 promotes the folding and stability of type II myosins and prevents their aggregation in *Caenorhabditis* *elegans* (5). In contrast with invertebrates, which have only one ubiquitously expressed UNC45 isoform, vertebrates have 2 isoforms that are only 55% identical and have a nonredundant function (4). UNC45B is exclusively expressed in striated muscle, binds to muscle type II myosins, and plays a key role in cardiac and or skeletal muscle development in mouse and zebrafish (6, 7). Accordingly, pathogenic variants in UNC45B were shown to cause myopathy in humans (8, 9). UNC45A is ubiquitous and was recently shown to control the folding of nonmuscle type II myosins and to regulate stress fiber assembly in U2OS osteosarcoma cells (10).

Based on our use of next-generation sequencing, combined with biochemical and functional validation, we describe the intestinal phenotype underlying biallelic variants in *UNC45A* in 6 patients from 5 unrelated families who presented with extremely severe CDD, mainly associated with cholestasis, deafness, and bone fragility. Through in silico and in vitro analysis, we investigated the mechanism underlying UNC45A dysfunction and showed that, in enterocytes, UNC45A participates in epithelial differentiation by promoting the folding of myosins, and notably of myosin VB. This unconventional myosin orchestrates apical trafficking and microvillus differentiation in enterocytes, and LoF *MYO5B* mutations are a well-known cause of microvillus inclusion disease (MVID) (11, 12). The generation of intestinal organoids from patients and the detailed characterization of the unc45a mutant in zebrafish provided a unique opportunity for unraveling what we believe to be a new mechanism of MVID.

## Results

### Next-generation sequencing identifies biallelic UNC45A variants in patients with congenital diarrhea.

**Figure 1:**
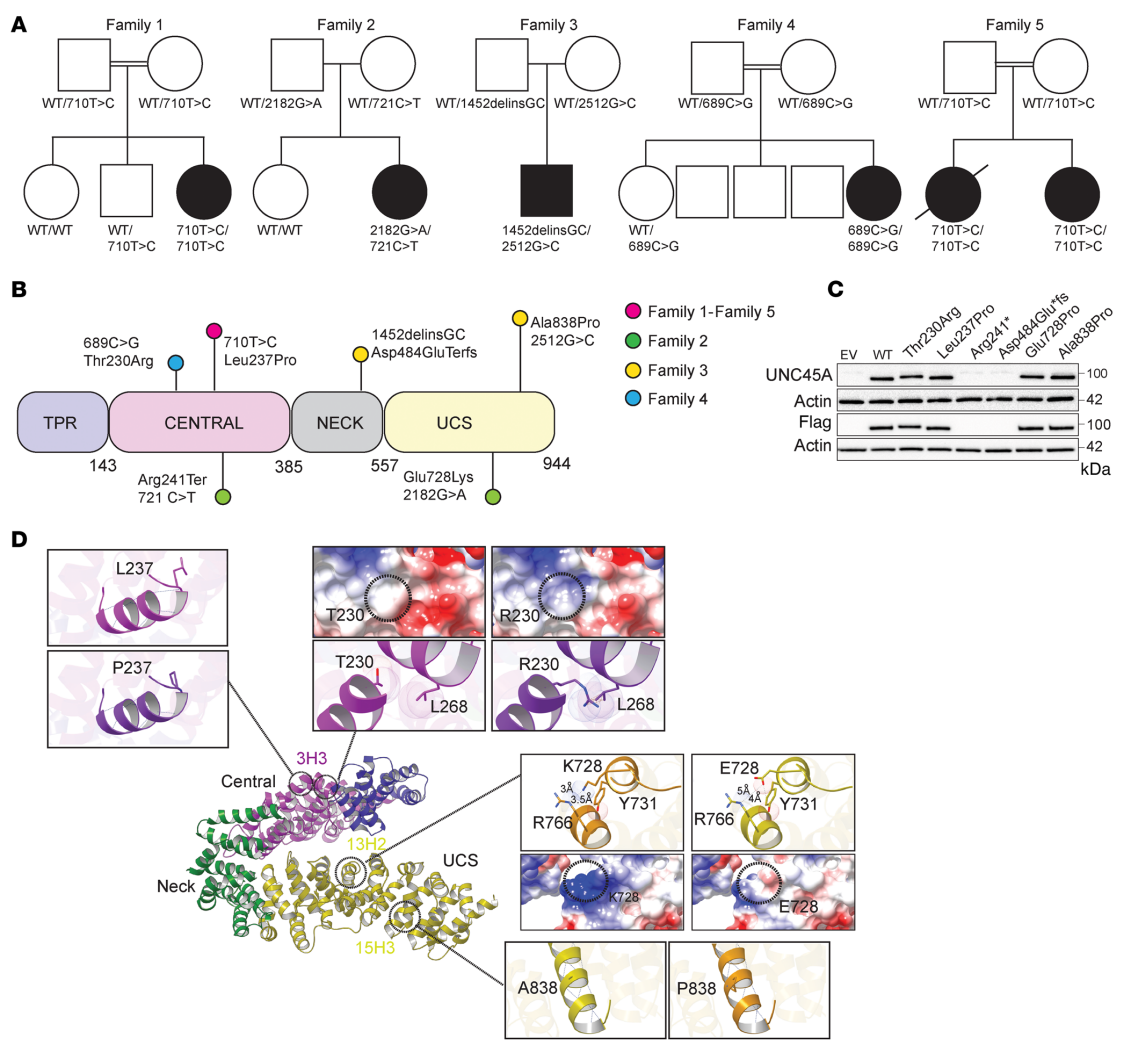
Genetics of 5 families with *UNC45A* deficiency and molecular characterization of UNC45A variants. (**A**) Pedigree of families with AR *UNC45A* deficiency (filled shapes indicate affected individuals). (**B**) Schematic representation of UNC45A protein showing location of the variants identified in this study. (**C**) Western blot analysis of UNC45A protein in HEK293T cells transfected with EV, WT, and mutant alleles (*n* = 2). (**D**) Ribbon representation of the human UNC45a model generated by MODELLER (based on the *D*. *melanogaster* and *C*. *elegans* UNC45 protein structures) showing its domain architecture. The variants were modeled in silico using Chimera. The location and a close-up view of each variant are shown. For the L237P and A838P mutations, hydrogen bonds are depicted by dark-blue dashed lines. For the T230R and E728K mutations, Van der Waals radii are represented for each atom and steric clashes occurring in the structure are shown with brown dashed lines. Coulombic surface representations are also shown. Red surface indicates the lowest electrostatic potential energy and blue the highest. Locations of the mutated residue are highlighted by a black circle. Distances are indicated using black dotted lines.

We investigated 6 patients from 5 unrelated families with CDD and intestinal failure associated or not with cholestasis, bone fragility, and deafness (Supplemental Table 1; supplemental material available online with this article; https://doi.org/10.1172/JCI154997DS1). To identify the underlying gene defect, whole-exome sequencing was performed for patient 1 (P1), P2, P3, and P5, while targeted panel sequencing was used in P4 and Sanger sequencing in P6. Variants in genes previously associated with congenital diarrhea and notably with MVID (*MYO5B*, *STX3*, *STXBP2*) were not identified. In contrast, rare biallelic variants in *UNC45A* (GenBank NM_018671.4) were found in all patients (Figure 1A). All variants were scored as damaging/deleterious by all prediction tools and associated with high combined annotation-dependent depletion (Supplemental Table 2). Familial segregation was confirmed by Sanger sequencing (Supplemental Figure 1A). Human *UNC45A* encompasses 23 exons spanning 4 kb and encodes a 944 aa protein with a 4-domain architecture comprising an N-terminal TPR domain (helix repeats 1–3; 1–143 aa), a central armadillo repeat (ARM) domain (helix repeats 1–5; 144–385 aa), a neck domain (helix repeats 6–9; 386–557 aa), and a C-terminal UCS domain (helix repeats 10–17; 558–950) (Figure 1B). P1 and siblings P5 and P6 carried a homozygous missense variant converting a leucine into a proline (c.710T>C, p.Leu237Pro) within the central domain. Compound heterozygous variants (c.721C>T, p.Arg241Ter and c.2182G>A p.Glu728Lys) in the central and UCS domain of UNC45A, respectively, were identified in P2. In P3, a 2 bp insertion/deletion led to an early stop codon (c.1452delinsGC, p.Asp484GlufsTer31), and a missense variant converted an alanine into a proline (c:2467G>C, p.Ala838Pro) within the UCS domain. P4 carried a homozygous missense variant (c.689C>G, p.Thr230Arg) within the central domain. All missense variants affected amino acids conserved among orthologs (Supplemental Figure 1B). Given the striking overlap between clinical symptoms in our patients and the partial overlap with recently described UNC45A-deficient patients, allele frequency, predicted pathogenicity, and familial segregation of the phenotype, we concluded that the observed variants in *UNC45A* were likely the cause of the disease.

### UNC45A variants impair protein expression or structure.

To address whether the variants affected UNC45A expression, all UNC45A variant alleles were ectopically expressed in HEK293T cells. No protein was detected for the Asp484GlufsTer31 and Arg241Ter alleles. The missense variants Thr230Arg, Leu237Pro, Glu728Lys, and Ala838Pro showed protein expression comparable to that of WT (Figure 1C). Similar results were obtained when the missense variants were overexpressed in UNC45A^KO^ Caco-2 cells (Supplemental Figure 2, A and B). In P1, this result was confirmed in T lymphoblastic cells (Supplemental Figure 2C). Yet while in control duodenal biopsies, UNC45A appeared localized both apically and intracellularly, loss of apical UNC45A was observed in P1 and P2 duodenal biopsies. In P2, UNC45A expression was also considerably decreased, likely the result of the allele Arg241Ter (Supplemental Figure 2D). To further evaluate the structural impact of the missense variants, the human UNC45A structure was built by homology modeling using the *C*. *elegans* and *D*. *melanogaster* UNC45 structures as templates (Protein Data Bank [PDB] entries: 4I2Z and 3NOW, respectively, with sequence identity close to 35%). Both Thr230 and Leu237 residues mapped in the helix 1 of the 3rd ARM repeat (3H1) of the central domain (Figure 1D). This domain connects the functional TRP domain that binds HSP70/90 partners and the UCS domain that mediates myosin binding (5). Replacement of Thr230 with a charged and bulkier Arg is predicted to alter surface charges and to induce steric hindrance with residues of the neighboring central domain helix 3H3 (notably the Leu268 residue) (Figure 1D). Similarly, replacing the conserved helix residue Leu237 with Pro most likely results in loss of a key backbone H-bond and in helix destabilization (13). As the helices of the central domain hold the TRP domain in place (14), by destabilizing central domain helix integrity, Thr230Arg and the Leu237Pro variants may alter the positioning of the TRP domain and thus protein conformation. The Glu728 and Ala838 residues localize in highly conserved motifs of 2 different helices (13H2 and 15H3) within the UCS domain (Figure 1D). The negatively charged Glu728 residue engages in ionic interactions with the Arg766 of a neighboring UCS helix. Therefore, its replacement with a positively charged residue such as Lys very likely induces electrostatic repulsion and surface charge alterations that may affect interactions between the 2 UCS helices. Exchange of the conserved helix residue Ala838 for a helix breaker residue such as proline most likely results in protein structure destabilization (13). These data suggest that the amino acid substitutions at the positions reported here are likely to lead to structural changes in UNC45A.

### Intestinal biopsies from UNC45A-deficient patients display an MVID phenotype.

**Figure 2:**
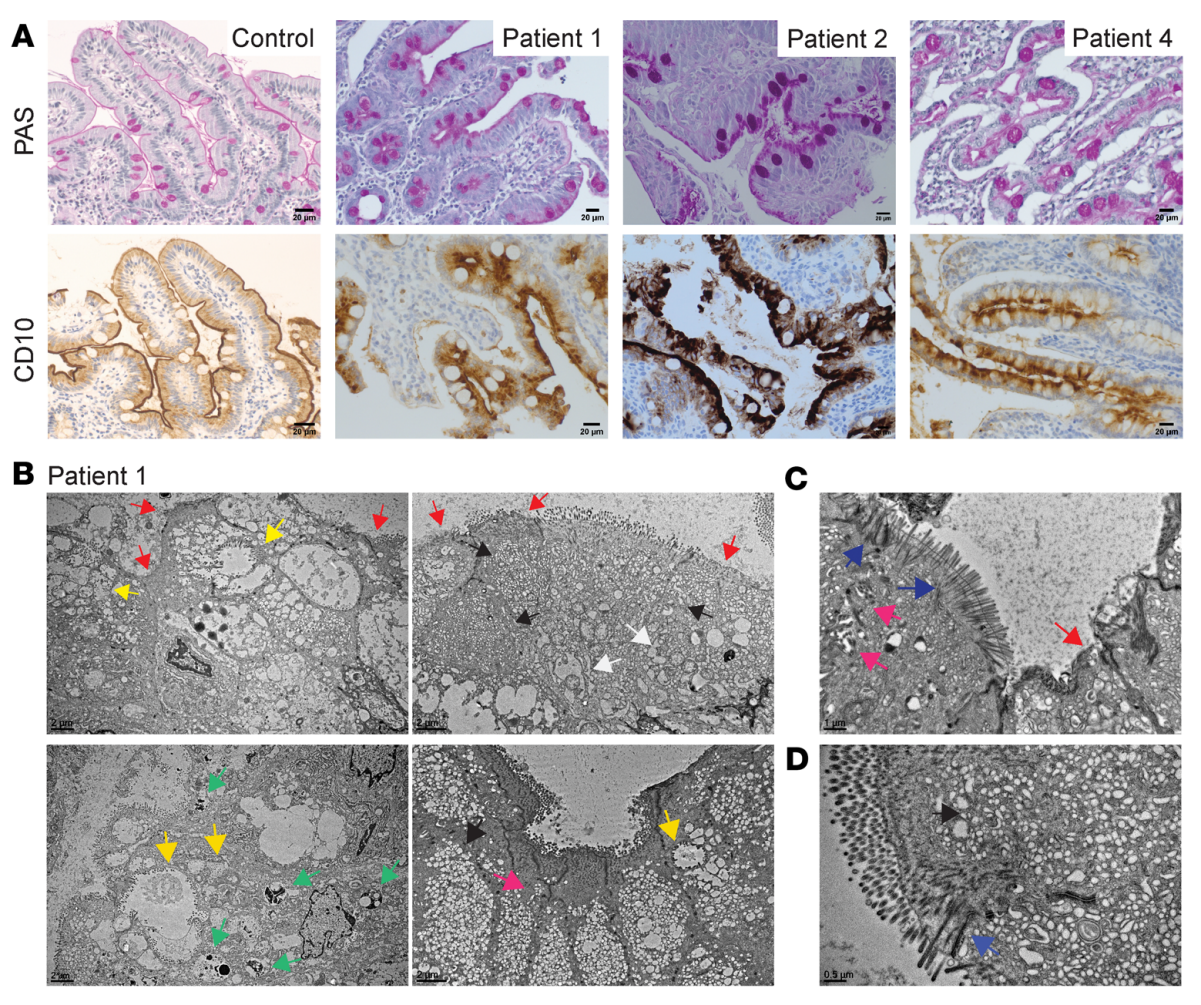
Histopathological and ultrastructural MVID features in the intestinal epithelium of patient. (**A**) PAS and CD10 staining in control, P1, P2, and P4 duodenal biopsies. Scale bars: 20 μm. (**B**–**D**) TEM of P1’s duodenal enterocytes showing ultrastructural features of MVID: regions containing vesicles and tubulovesicular structures (black arrowheads), enlarged lysosomes (green arrowheads), and swollen (i.e., stressed) endoplasmic reticulum (white arrowheads) as well as more or less complex or partially degraded MVIs (yellow arrowheads) and basolateral microvilli (pink arrowheads). Brush border showed defective microvilli anchored deep into the cytoplasm and thickened terminal web (blue arrowheads) or partially depleted microvilli (red arrowheads). Scale bars: 2 μm (**B**); 1 μm (**C**); 0.5 μm (**D**).

Duodenal biopsies were available from patients P1, P2, and P4. They all showed villus atrophy with no inflammation (Supplemental Figure 3A). Yet subapical accumulation of CD10 and diffuse periodic acid–Schiff (PAS) staining were strongly reminiscent of MIVD (ref. 15 and Figure 2A). Electron microscopy (EM) of P1 and P2 small intestine showed the full spectrum of subcellular features of MVID (Figure 2B, Supplemental Figure 3, B and C, and Supplemental Figure 4), supporting this assumption. Both villus and crypt enterocytes showed local malformation, reduction, or complete erosion of apical microvilli and subapical accumulation of pleomorphic vesicles, including abundant PAS-positive “secretory granules” (16) as well as ectopic microvilli forming either microvillus inclusions (MVI) of variable morphology or occurring basolaterally. Basolateral microvilli were an unusual feature of crypt enterocytes that appeared structurally more complex (Supplemental Figure 4, H–J) than the slightly bent, finger-like protrusions described previously (17, 18). Groups of irregularly arranged, straight microvilli with distinct actin core and long rootlets deeply anchored in a terminal web-like zone almost devoid of organelles regularly occurred in close proximity to the basement membrane and along the lateral surface of enterocytes. Similar abnormal microvilli deeply anchored in the terminal web also characterized the disorganized apical brush border areas of both patients (Figure 2, B and C, and Supplemental Figure 3, B and C) in contrast to the uniform packing of structured microvilli observed in controls (Supplemental Figure 3, D and E). Finally, lysosomal organelles were remarkably larger in villus enterocytes with UNC45A deficiency (Supplemental Figure 4C), but normal in crypts (Supplemental Figure 4F). Overall, histology of UNC45A-deficient intestinal biopsies qualifies this disorder as a variant of MVID.

### UNC45A contributes to epithelial cell polarity, differentiation, and lumen formation.

**Figure 3:**
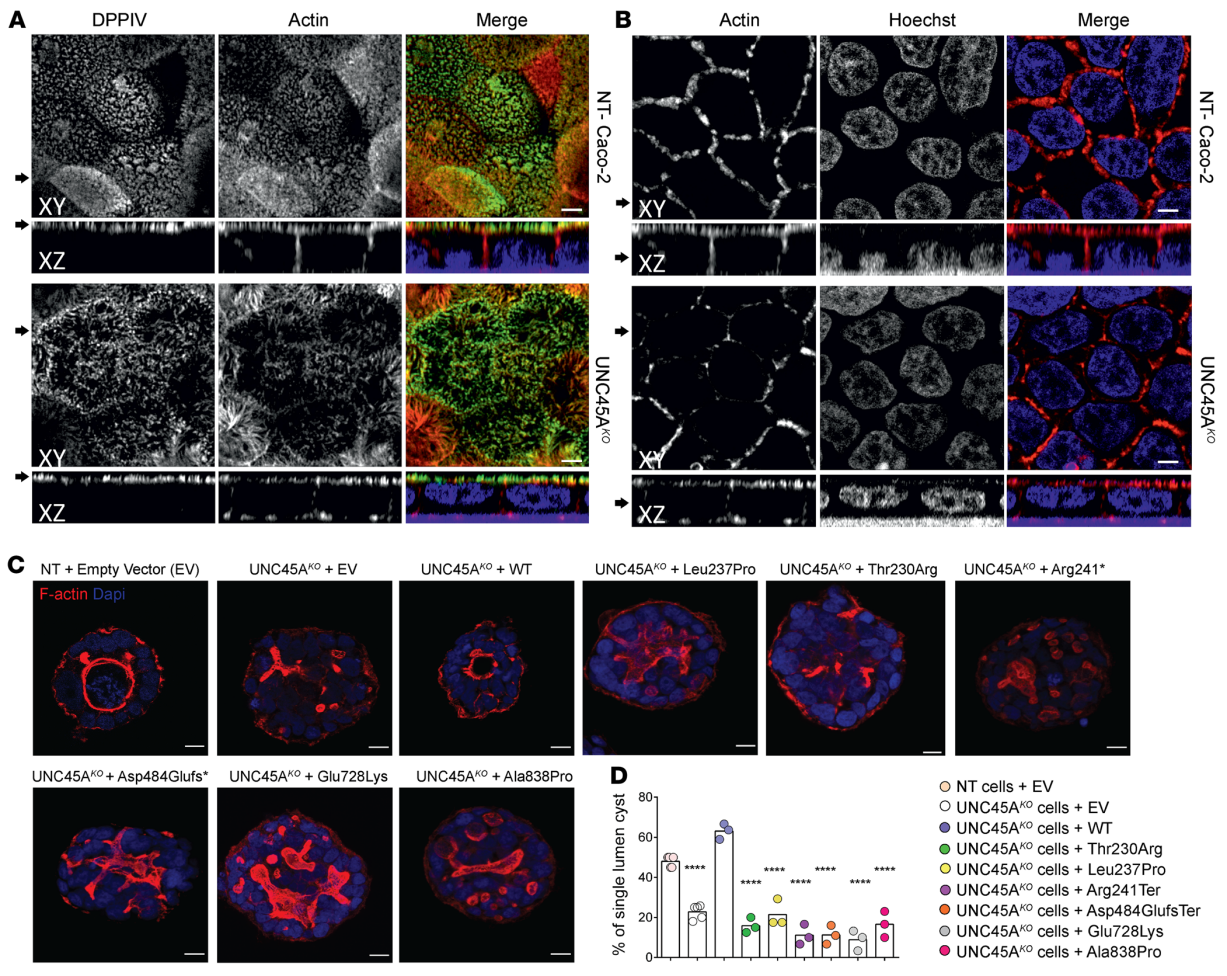
Disrupted enterocyte architecture in *UNC45A* deficiency. (**A**) Confocal images of polarized NT control and Unc45A^KO^ Caco-2 cells grown on a filter and stained for an apical brush border marker DPPIV and actin. (**B**) Actin staining in polarized Caco-2 cells. Nuclei were visualized with HOECHST. Arrows on the left mark the corresponding XY and XZ planes. Scale bars: 20 μm. Panels **A** and **B** were from the same experiment. (**C**) NT control and UNC45A^KO^ Caco-2 cells complemented or not with WT or mutant alleles were cultured in 3D for 5 days to form cysts. Nuclei are stained with Nucblue (blue); actin is stained with phalloidin AF 455 (red). Single confocal sections through the middle of the cyst are shown. Scale bars: 10 μm. (**D**) Single-lumen cysts were counted in each experiment. Results from 3 independent experiments (35 cysts each) are shown, 1-way ANOVA. ****P < 0.0001.

To assess whether loss of UNC45A could affect enterocyte polarity and differentiation and thereby contribute to the MVID-like phenotype, we first knocked out UNC45A in the enterocyte-like Caco-2 cell line by CRISPR/Cas9-mediated genome editing. UNC45A inactivation compared with nontargeting (NT) control line was confirmed by immunoblot (Supplemental Figure 2A). Immunofluorescence staining for the apical marker DPPIV and for actin revealed that depletion of UNC45A reduced the density and caused morphological irregularities of apical brush border without other major changes in actin organization in polarized Caco-2 cells on filters (Figure 3, A and B). We then switched to 3D culture in Matrigel, a well-known model of epithelial morphogenesis (19). As expected, NT Caco-2 cells in Matrigel organized within 5 days into hollow, round cyst structures made of a monolayer of cells with the apical surface oriented toward a single central lumen lined by F-actin. In contrast, UNC45A^KO^ Caco-2 cells largely failed to develop well-defined lumens and instead formed cysts with multiple small cavities lined by patchy accumulation of F-actin (Figure 3C). While complementation of UNC45A^KO^ Caco-2 cells by WT-UNC45A significantly increased the number of cysts with well-formed lumen, all mutant alleles failed to do so (Figure 3, C and D). Overall, these findings highlighted the key role of UNC45A in controlling the organization of the apical pole and lumen formation in enterocytes and showed that the mutant UNC45A proteins failed to perform this function. Moreover the defect in lumen formation in UNC45A^KO^ cysts mimicked the one reported in MYOVB^KO^ cysts (20), suggesting a possible functional link between UNC45A and myosin VB.

### Myosin VB binds UNC45A and is misfolded in UNC45A-deficient conditions.

**Figure 4:**
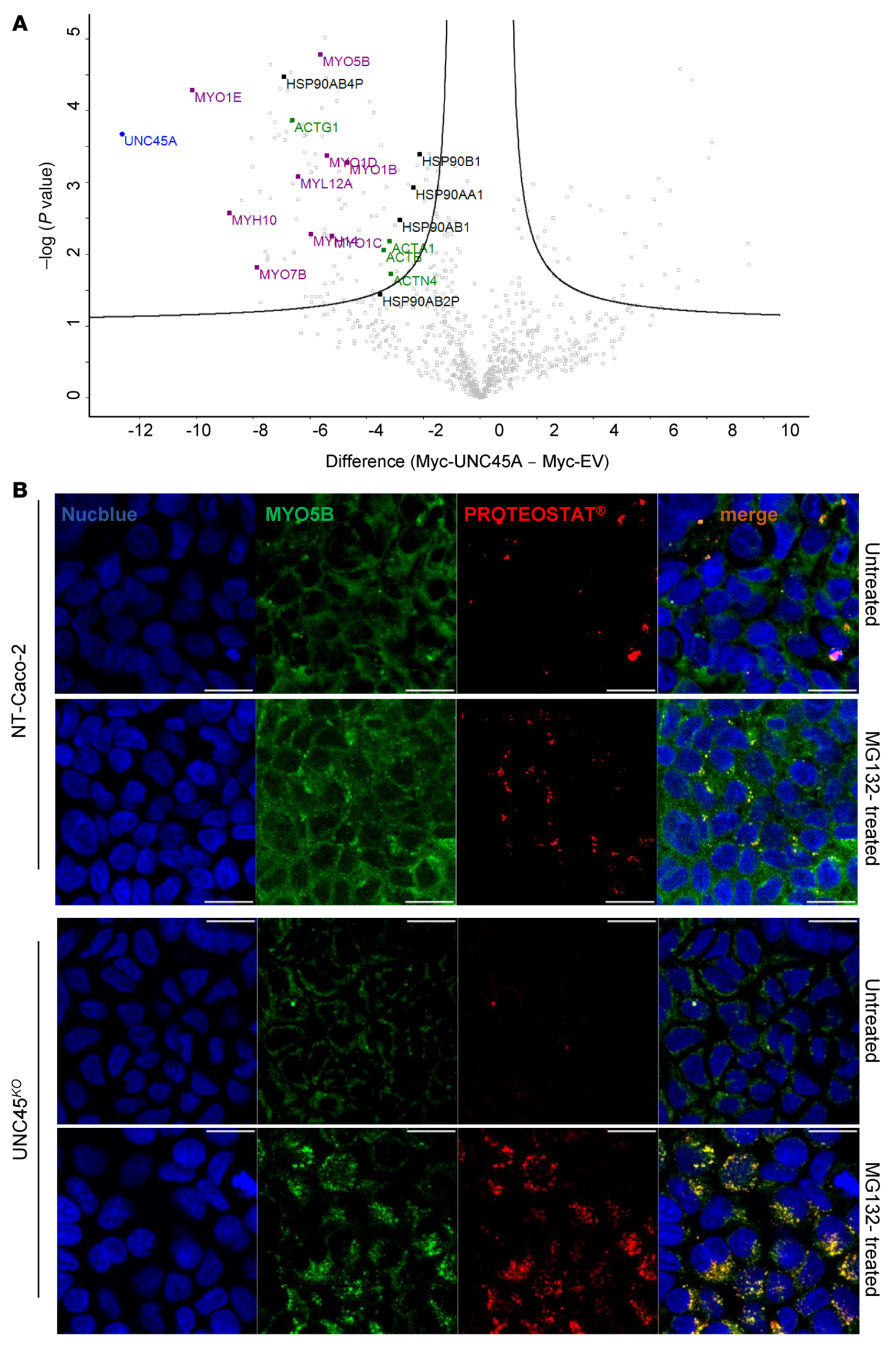
MYO5B as a client of the HSP90-UNC45A chaperone complex. (**A**) Volcano plot showing proteins enriched in the myc-tagged UNC45A WT immunoprecipitates over the myc-tagged EV control identified by mass spectrometry in 3 independent pull-down assays. The difference of the average of the logarithm of Label Free Quantification (LQF) intensities (WT vs. EV) is plotted against negative logarithmic *P* values of a 2-sided, 2-sample Welsh *t* test. The hyperbolic curve delimitates significantly enriched proteins from common hits. High LFQ hits of interest are indicated in black (HSP90 family), in purple (Myosin protein family), and in green (Actin protein family). (**B**) Accumulation of myosin VB aggregates (aggresomes) in UNC45A^KO^ cells treated with MG-132 (10 μM) overnight. Scale bars: 10 μm. *n* = 3. Nuclei are stained with Nucblue (blue).

To delineate which myosins are the clients of the UNC45A-HSP90 chaperone complex in enterocytes, lysates from myc-tagged UNC45A Caco-2 cells and myc-tagged empty vector (EV) control Caco-2 cells were immunoprecipitated with myc-Ab and pulled-down proteins were comparatively analyzed using Orbitrap mass spectrometry. 111 Proteins were coimmunoprecipitated exclusively in Caco-2 cells transduced with myc-tagged UNC45A (Supplemental Table 3). The proteins most significantly enriched in the WT UNC45A precipitate included 4 HSP90 proteins, α and β actins, and 9 myosins, including several nonconventional myosins, notably myosin VB (Figure 4A). The interaction of WT-UNC45A with myosin VB and HSP90 was confirmed by Western blot (Supplemental Figure 5A). Strikingly, treatment of UNC45^KO^ Caco-2 cells with the proteasome inhibitor MG132 resulted in the appearance of large myosin VB–positive aggregates (Figure 4B), indicating that UNC45A is critical for preventing aggregation and incorrect folding of myosin VB, which overall suggests that UNC45A deficiency could phenocopy the MVID phenotype.

### UNC45A^KO^ and patient-derived organoids recapitulate in vivo findings.

**Figure 5:**
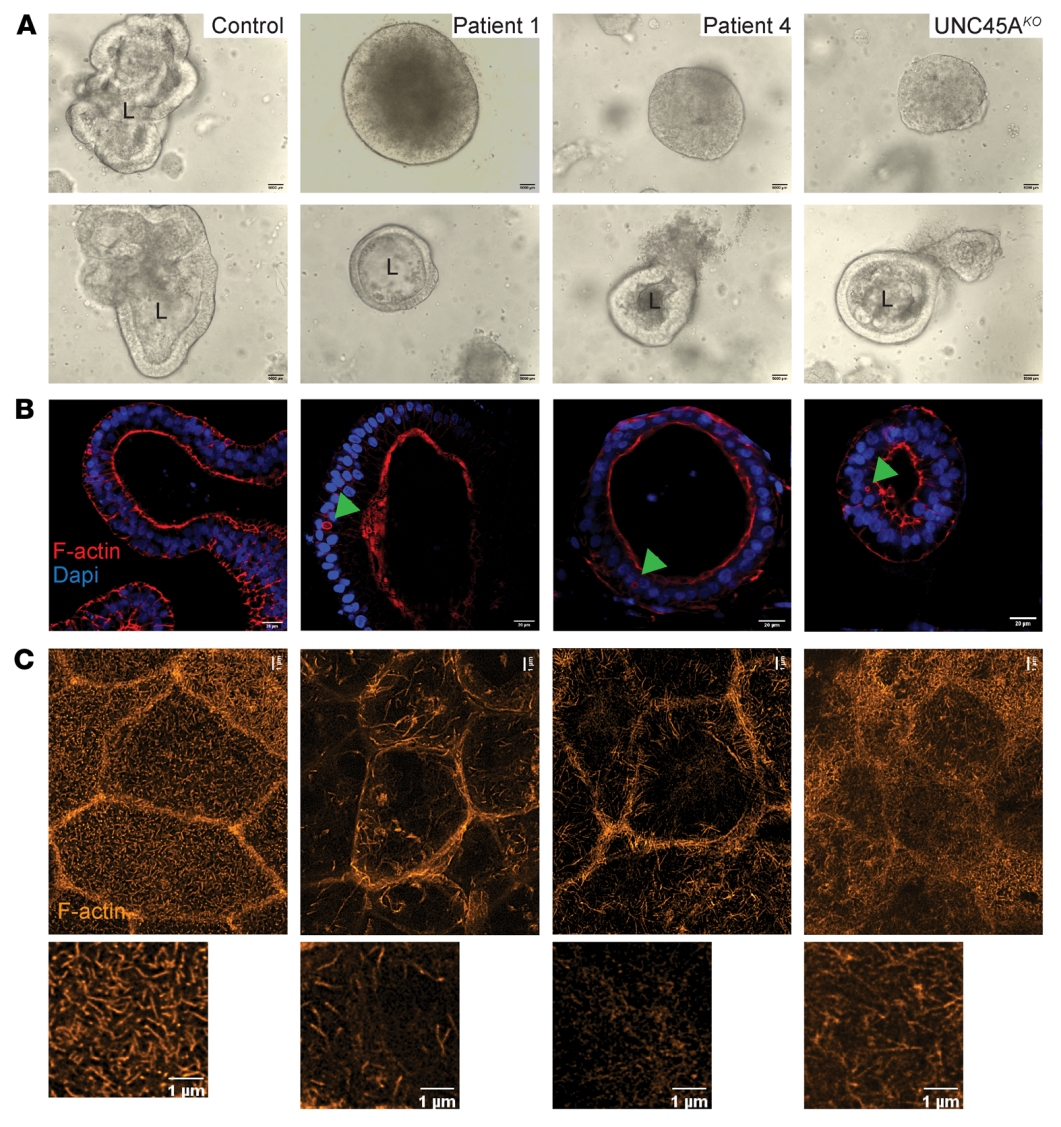
UNC45A-deficient organoids recapitulate MVID features. (**A**) Bright-field images of control, P1, P4, and UNC45A^KO1^ 3D organoids. Scale bar: 5000 μm. (**B**) F-actin staining revealing MVIs in the small subset of P1, P4, and UNC45A^KO^ 3D organoids that display a central lumen. Scale bar: 20 μm. (**C**) STED image of F-actin at the apical pole of 2D enterocyte cultures derived from control, P1, P4, and UNC45A^KO1^ organoids after a 21-day culture on filters. Scale bars: 1 μm. L, lumen.

**Figure 6:**
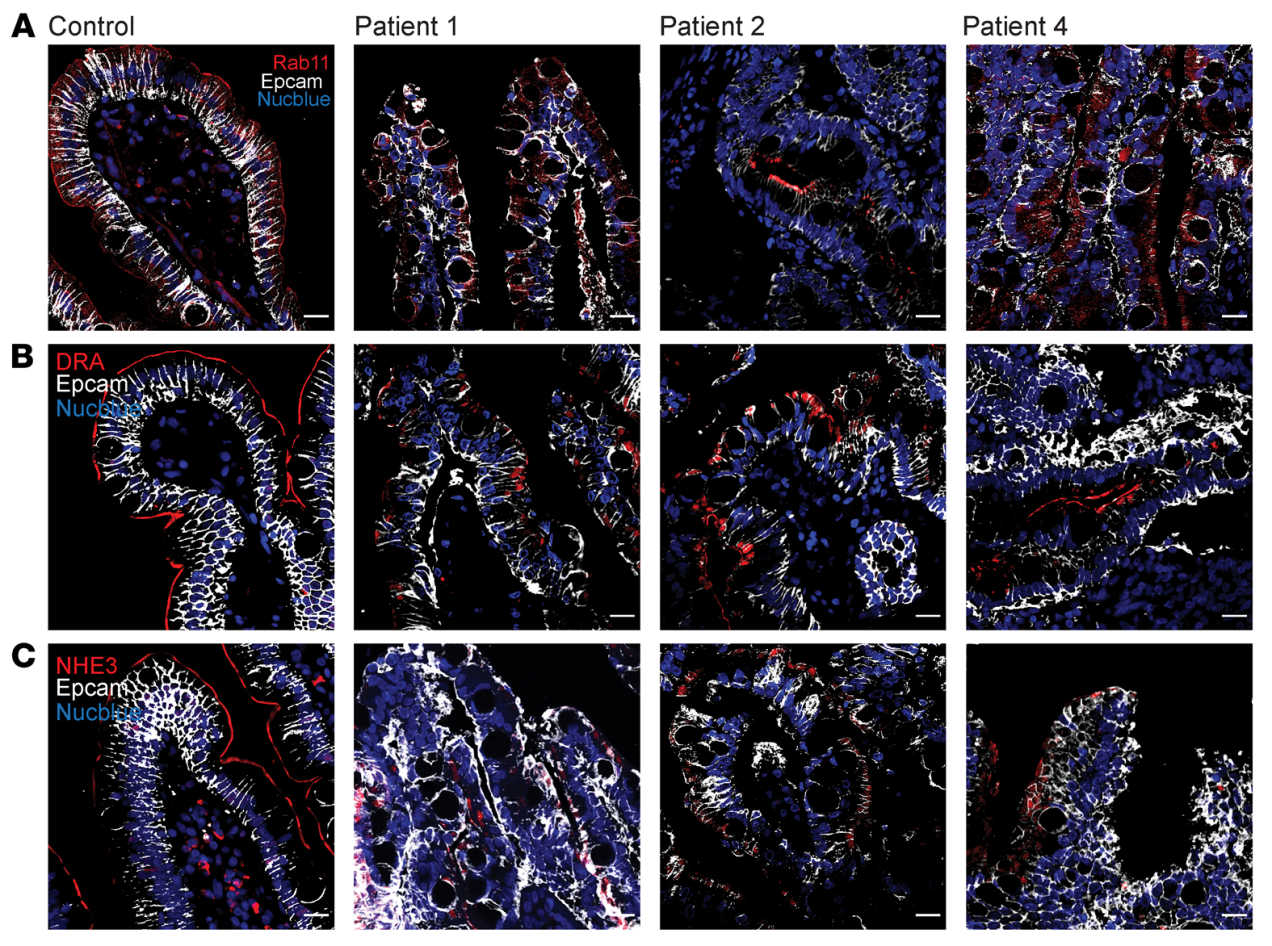
Impaired apical targeting in UNC45A-deficient patients. (**A**–**C**) Confocal imaging showing localization of Rab11 (**A**) and of apical transporters DRA (**B**) and NHE3 (**C**) in duodenum of control and patients. Scale bars: 20 μm.

To establish a physiological model of epithelial differentiation, 3D organoid cultures were generated from 2 controls and from patients (P1, P4) using blood-derived induced pluripotent stem cells (iPSCs). iPSC-derived intestinal organoids retain a fetal intestinal phenotype characterized by incomplete maturation of the brush border (21). However, this technique has been used to overcome ethical concerns and difficulties arising from the need to take biopsies from sick infants. UNC45A-knockout organoids (UNC45A^KO^) were obtained by CRISPR/Cas9 genome editing of control iPSCs (Supplemental Figure 5B). In contrast to control organoids, the majority of patient organoids showed abnormal intestinal architecture characterized by dense cellular aggregates deprived of central lumen (villus domain) and lacking crypt budding (Figure 5A and Supplemental Figure 5C), overall mirroring the phenotype of UNC45A^KO^ Caco-2 cysts. This gross morphology was recapitulated in UNC45A^KO^ organoids (Figure 5A). Yet about 30% of patient-derived or UNC45A^KO^ organoids developed a single lumen (Figure 5, A and B, and Supplemental Figure 5C) and, strikingly, some of them showed large F-actin–positive intracellular inclusions (Figure 5B). Transmission electron microscopy (TEM) analysis confirmed the presence of large MVI in the cytoplasm of enterocytes in both UNC45A^KO^ and patient organoids (Supplemental Figure 5D). MVI are hallmarks of MVID and have notably been observed in patients with variants that disrupt myosin VB interaction with the GTPase RAB11, which is crucial for trafficking between apical recycling endosomes and the apical membrane (22). To further study the putative role of UNC45A in myosin VB–dependent apical trafficking, organoids were adapted to 2D Transwell culture. While Rab11 showed the expected subapical vesicle localization in control organoids, its expression decreased and it localized away from the apical side in both UNC45A^KO1^ and patient organoids (Supplemental Figure 6A). Importantly, similar mislocalization and reduced expression of Rab11 were observed in duodenal tissues from UNC45A-deficient patients (Figure 6A), confirming impaired apical trafficking in vivo. One well-established consequence of impaired apical trafficking in MVID caused by *MY05B* is the loss of apical expression of NHE3, the Na^+^-H^+^ exchanger, and of DRA, the chloride bicarbonate exchanger, both of which participate in intestinal regulation of water and solute absorption (23). While 2D organoid cultures derived from control iPSCs formed a well-organized monolayer with apical localization of NHE3 and DRA, 2D cultures from P1 and P2 and UNC45^KO^ organoids were less well organized, with intracellular localization of NHE3 and DRA (Supplemental Figure 6, B and C). Similarly, these transporters were strongly and exclusively localized at the apical brush border of villus enterocytes in control duodenal tissues, while their expression was decreased and exclusively intracellular in UNC45A-deficient patients (Figure 6, B and C). In contrast and consistent with the results in MVID due to MY05B deficiency, cystic fibrosis transmembrane conductance regulator (CFTR), which facilitates cyclic AMP-dependent chloride transport, showed comparable apical distribution in 2D organoid cultures derived from control and from UNC45A-deficient iPSC lines (Supplemental Figure 6D). In addition, comparable swelling of control and UNC45A-deficient 3D organoids upon stimulation by forskolin demonstrated normal CFTR-mediated apical secretion (Supplemental Figure 7, A and B). Further in line with findings in MVID, E-cadherin, integrin-α2, and Na/K-ATPase displayed normal basolateral membrane distribution in the duodenal tissue from patients (Supplemental Figure 8A) as well as in UNC45A-deficient monolayers (Supplemental Figure 8, B–D), indicating normal basolateral trafficking. Overall, lack of apical Na^+^ transporters and retention of CFTR at the apical pole of enterocytes suggest that the inability to absorb Na^+^ combined with active Cl^–^ secretion may be the driving cause of diarrhea in UNC45A deficiency. Finally, super-resolution stimulated emission depletion (STED) imaging of the apical pole of 2D organoid cultures revealed that organization of microvilli was severely impaired in UNC45-deficient conditions with notably significantly reduced density (Figure 5C and Supplemental Figure 5E). Together, these results largely recapitulated observations made in myosin VB–deficient patients as well in *myo5b^KO^* mice (23) and pigs (24).

### Enterocytes in unc45 zebrafish mutants display MVID features.

**Figure 7:**
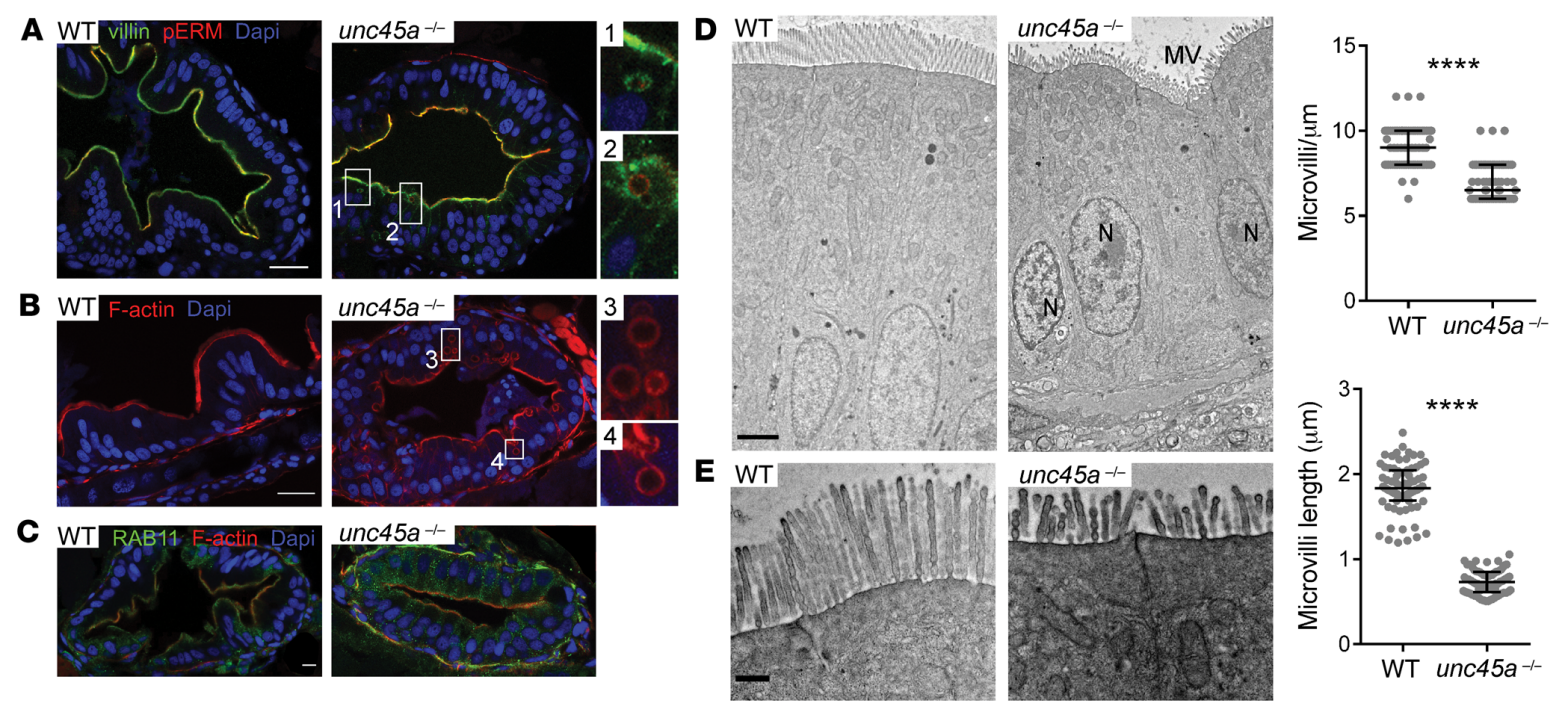
MVID features in enterocytes of *unc45a* zebrafish mutants. (**A**–**C**) Confocal microscopy analysis of the intestinal bulb of WT and *unc45a^–/–^* mutant larvae stained for villin and pERM (**A**), F-actin (phalloidin) (**B**), and Rab11 (**C**) at 5 dpf. Scale bars: 20 μm. Boxes showing microvillus-like inclusions are enlarged ×4. (**D** and **E**) TEMs of thin sections of intestinal bulb of 5 dpf WT and *unc45a^–/–^* mutant larvae showing defects in the organization of the brush border. Scale bars: 2 μm (**D**); 0.5 μm (**E**). Quantitative analysis showing decrease in microvillus length and density in *unc45a^–/–^* enterocytes as compared with WT enterocytes. Data are represented as mean + SD. ****P < 0.001, *t* test. N nucleus; MV microvilli.

We next explored UNC45A function in gut morphogenesis in vivo using the previously described zebrafish line (25). Loss of epithelial folds was confirmed in *unc45a* mutant larvae (ref. 3, Supplemental Figure 9, and Figure 7, A–C), a phenotype also reported in *myo5b*^KO^ zebrafish (26). In both 5 dpf WT and mutant larvae, strong staining of villin lined the enterocyte apical brush border (Figure 7A). Yet strikingly, *unc45a^KO^* enterocytes showed the accumulation of large villin-positive compartments (Figure 7A) absent in WT larvae. These compartments were also detected by phalloidin staining and were mostly localized under the apical membrane or close to the apical pole and also stained internally for phospho–Ezrin-Radixin-Moesin (phospho-ERM), suggesting that they might result from invagination of the apical membrane (Figure 7A). These structures were also reported in *myo5b^KO^* zebrafish (26). Furthermore, while Rab11-positive vesicles localized just beneath the apical cell surface in WT enterocytes, those vesicles mislocalized to the basolateral surface of intestinal cells in *unc45a^KO^* mutant larvae (Figure 7C). The lack of antibodies crossreacting with NHE3/DRA zebrafish proteins prevented the evaluation of their localization in mutant larvae. However, TEM analysis of the intestines of larvae at 5 dpf showed that microvilli were significantly shorter and less uniformly packed in *unc45a^KO^* enterocytes compared with WT larvae (Figure 7, D and E). Overall, these data suggest that the *unc45a* mutant accurately reflects aberrant features of MVID, with a marked accumulation of vesicles and tubulovesicular structures as well as a reduction in microvillus length and density.

## Discussion

The development and the maintenance of polarity in intestinal epithelial cells with functionally distinct apical and basolateral domains are key for barrier formation and for nutrient absorption. Monogenic disorders causing aberrant trafficking of apical and basolateral proteins or of vesicular membrane compartments result in MVID (11, 18, 27). Recently, osteo-oto-hepato-enteric (O2HE) syndrome, presenting with severe CDD, severe liver cholestasis, deafness, and bone fragility, was attributed to *UNC45A* deficiency (3). Here, we report 6 additional patients carrying *UNC45A* variants associated with similar patient phenotypes. Despite the modeling of *UNC45A* deficiency in zebrafish (3), the mechanism underlying the intestinal phenotype and the overall function of UNC45A in this tissue has remained unclear. Herein, we have taken advantage of the identification of as-yet-undescribed *UNC45A*-deficient patients with severe diarrhea to mechanistically dissect the role of UNC45A in the gut epithelium. In-depth histological characterization of intestinal biopsies from 3 patients provided decisive clues to the role of UNC45A in enterocyte polarization. Erosion of the brush border, accumulation of subapical PAS-positive vesicles, the presence of ectopic microvilli (within MVI and basolaterally), and enlarged lysosomal organelles observed in duodenal biopsies from patients are all typical features of MVID, an autosomal recessive disorder known to be caused by variants in *MYO5B* (11), *STX3* (18), and *STX2BP* (27), 3 genes encoding functionally and spatially related proteins that coordinate apical membrane trafficking in enterocytes. Most variants affect *MYO5B*, which encodes the actin-based molecular motor myosin VB (11). By interacting with the Rab11 GTPAse, myosin VB allows the docking and transport of cargo vesicles and proteins along actin fibers toward the enterocyte apical surface, including, notably, transporters involved in absorptive function (23, 24). In this study, proteomic analysis of UNC45A interactome identified myosin VB as a client of UNC45A. We further showed that UNC45A forms a stable complex with the chaperone Hsp90 in intestinal epithelial cells and acts as a critical cochaperone for myosin VB to promote its proper folding. Accordingly, propensity to aggregation of myosin VB was drastically increased in UNC45A^KO^ Caco-2 cells. Recapitulating observations in *MYO5B*-deficient conditions (20), UNC45A^KO^ Caco-2 cells and organoids largely failed to form cysts with single central lumen. Moreover, UNC45A-deficient organoids displayed MVI and impaired myosin VB–dependent targeting of NHE3 and DRA at the apical membrane while STED microscopy disclosed abnormal microvilli formation. As described under *MYO5B*-deficient conditions, apical localization and function of CFTR were not affected by loss of UNC45A and basolateral localization of E-cadherin, Na/K ATPase, and integrin-α2 were also preserved (24). The striking overlap between the consequences of *UNC45A* and of *MYO5B* deficiency were further emphasized by observations in the zebrafish model initially reported by Esteve et al. (3). These authors had pointed to a defect in intestinal motility, but they also reported reduced epithelial folds in the proximal intestine (3). Herein, we confirm and extend these results by showing that the zebrafish *unc45a* mutant recapitulates features of MVID, notably microvillus shortening and MVI-like compartments as well as Rab11 mislocalization. Strikingly, similar patterns were also reported in *MYO5B*-deficient zebrafish (26).

In conclusion, our results unravel the central role of UNC45A in shaping enterocyte architecture and polarization and show that UNC45A deficiency should be considered as a nonclassical form of MVID. Our data obtained from several complementary approaches demonstrate how the study of rare genetic diseases can provide insights into the mechanisms that control human intestinal barrier differentiation.

## Methods

### Patients.

P1 (girl) was the third child of Turkish first-degree cousins. Small bowel and colon dilatation were first detected by ultrasonography in the seventh month of pregnancy. At birth, 5 weeks before term, her low weight (2100 g) attested to intrauterine growth retardation. She developed severe protracted diarrhea, which caused hypovolemic shock at 3 weeks of age. Total parenteral nutrition (TPN) failed to stop diarrhea, and total enterectomy was performed at 20 months in order to control hydroelectrolytic balance. At this writing, the patient is 4 years old and remains on 12 hours of cycling parenteral nutrition. P2 (girl) was born of a nonconsanguineous family of British origin without family history of congenital diarrhea. At 1 week, she presented with severe protracted diarrhea requiring TPN. P3 (boy) was the only child of a French nonconsanguineous family born at term with low weight and height. Severe diarrhea was diagnosed 1 week after birth, leading to dehydration associated with hepatic cholestasis requiring long-term hospitalization with exclusive enteral nutrition. Diarrhea became intermittent until age 10 and then stopped. At this writing, he is 21 years old. P4 (girl) was the fifth child of a French consanguineous family with West Indies origins. Severe protracted diarrhea started at 4 days after birth. She initially received TPN, then underwent small bowel transplantation. P5 and P6 were the 2 children of Turkish first-degree cousins. P5 (girl) was born small for gestational age at 35 weeks. She was treated for suspected septicemia because of lethargy and failure to thrive, icterus, metabolic acidosis, and watery diarrhea (7–10 times/day) since birth. She died from multiorgan failure at 3 months of age. Her younger sister, P6, also showed failure to thrive, icterus, and watery diarrhea since birth and was TPN dependent at age 3 months. P1, P3, and P4 presented with extragastrointestinal manifestation (Supplemental Table 1).

### DNA sequencing.

Next-generation and conventional Sanger sequencing were performed on DNA extracted from peripheral mononuclear cells as previously described (3, 28, 29).

### Western blot, immunoprecipitation, and proteomic studies.

For Western blot, cells were lysed in RIPA buffer (Sigma-Aldrich) supplemented with cOmplete protease inhibitors (Roche, Sigma-Aldrich). Protein concentration was measured by Bradford protein assay (Bio-Rad). Twenty micrograms of proteins in Laemmli buffer (Bio-Rad) were separated by SDS–PAGE, transferred to a polyvinylidene difluoride membrane (Bio-Rad), blocked with 5% milk protein in TBST (0.5% Tween, Bio-Rad), and probed with primary antibodies. The membranes were washed with TBST and incubated with appropriate secondary antibodies. For immunoprecipitation, cells were lysed in buffer (10 mM Tris/HCl, 150 mM NaCl, 0.5 mM EDTA, 0.5% NP40) with cOmplete protease inhibitors (Roche, Sigma-Aldrich), and myc-tagged proteins were immunoprecipitated by using the μMACS c-myc Isolation Kit (Miltenyi Biotec) according to the manufacturer’s instructions. For proteomic studies, peptide processing of myc-tagged immunoprecipitates and data analysis were performed as previously described (30).

### Generation of UNC45A-deficient Caco-2 cells and complementation with WT and mutant UN45A alleles.

UNC45A^KO^ Caco-2 cells were generated by CRISPR/CAs9-mediated genome editing (31). Two single guides (sg 1: 5′-CACCGATGTCAAAGCACTCTACCGG-3′ and sg2: 5′-CACCGATGTCAAAGCACTCTACCGG-3′) targeting UNC45A were designed with the CRISPR design tool (https://www.benchling.com/) and cloned into the lentiCRISPRv2 vector (52961, Addgene). A control NT guide (5′-GGACAATCATGGTGAAAGCGG-3′) was used as described (31). Lentiviral particles were produced by transfecting HEK293T cells with transfer plasmid, packaging psPAX2 (12260, Addgene), and VSV-G envelope PMD2.G (12259, Addgene) expressing plasmids using Lipofectamine 2000 (Invitrogen, Thermo Fisher Scientific) according to the manufacturer’s instructions. The recombinant virus–containing medium was filtered and used to transduce Caco-2 cells in the presence of polybrene (4 μg/mL) (Sigma-Aldrich). Positively transduced cells were selected with puromycin (Gibco, Thermo Fisher) at a concentration of 10 μg/ml. For complementation studies, human UNC45A (NM_018671.3, RC200151L1, Origene) was subcloned in pLVX-EF1α-IRES-mCherry vector (631987, Clontech). To introduce the different mutations, the GENEART Site-Directed Mutagenesis System (Invitrogen) was used according to the manufacturer’s instructions. Each construct was fully sequenced after mutagenesis. UNC45A-KO Caco-2 cells were transduced by lentiviral particles coding WT, mutant alleles, or EV. mCherry-expressing cells were sorted by flow cytometry (SH800S Cell Sorter, Sony) and expanded in culture.

### Cell culture.

Caco-2 cells (HTB37, 60143947, ATCC) were cultured in DMEM (Gibco, Thermo Fisher Scientific) with 10% FCS (Gibco, Thermo Fisher Scientific). For some experiments, the proteasome inhibitor MG132 (Sigma-Aldrich) was used overnight at a concentration of 10 μM. For 3D culture, Caco-2 cells were resuspended at a concentration of 104 cells/mL in medium containing 4% Matrigel (BD Biosciences), and 250 μl was plated per well in an 8-well chamber slide ibidi (Biovalley), precoated with 100 μL of Matrigel, and left 10 minutes at 37°C for solidification; 150 μl of medium was added. Cells were grown for 5 days to obtain cysts. For immunofluorescence studies, cysts were fixed in 4% PFA 30 minutes at 37°C and washed with PBS before incubation with antibodies.

### Immunofluorescence and imaging.

Paraffin sections (5 μm) were dewaxed in xylene and rehydrated in graded alcohol followed by 2 washes in PBS. Heat-mediated antigen retrieval was achieved in Target Retrieval Solution citrate buffer (Dako, Agilent Technologies). Nonspecific binding was blocked by 2% normal donkey serum (Sigma-Aldrich) in PBS for 45 minutes at room temperature (RT). Cysts or organoids were permeabilized with PBS with 0.1% Triton X-100 or with PBS with 2% Triton X-100 and then blocked in 3% BSA for 30 minutes. After 3 washes in PBS, cysts or organoids were incubated with primary antibodies diluted in Antibody Diluent Solution (Life Technologies, Thermo Fisher Scientific) overnight at 4°C. Secondary antibodies diluted in Antibody Diluent Solution were added after 3 washings (15 min/each) for 1 hour at RT. The antibodies used are listed in Supplemental Table 1. Mounting medium Mowiol (Sigma-Aldrich) was added on 3D cultures. Imaging was performed on a confocal microscope (TCS SP8, Leica Microsystems), and images were acquired with a 40 × 1.3 objective (HC PL APO, oil immersion, Leica Microsystems) and analyzed with ImageJ software from Fiji (https://imagej.nih.gov/ij/index.html). STED imaging was performed using a LEICA SP8 gSTED 2D microscope with an objective HC PL APO CS2 100X/1.4 oil immersion and white light laser with the following acquisition parameters: excitation, 558 nm; depletion, 660 nm, hybrid detector with gating. To improve the signal-to-noise ratio, the images were deconvolved with Huygens Professional Software (Scientific Volume Imaging) using the fast deconvolution presettings. The percentage of area covered by microvilli/cell was measured with ImageJ. Immunofluorescence analysis of Caco-2 cells grown on Transwell filters was performed as described previously (18). Single confocal planes or stacks from samples mounted in Mowiol were recorded with a confocal fluorescence microscope LSM980 (Zeiss) using a glycerol 63× lens with a numerical aperture of 1.3. The recording and deconvolution software used was ZEN3.3 (Zeiss). ImageJ software was used for scaling, brightness, and contrast adjustment.

### Antibodies and probes.

For immunofluorescence, antibodies and the probes used were as follows: F-actin (phalloidin–Alexa Fluor 488, catalog A12379 or phalloidin–Alexa Fluor 647, catalog A22287, Invitrogen); nuclei (NucBlue Fixed Cell Stain Ready Probes, catalog R37606, Invitrogen); aggresome (ProteoStat, catalog ENZ-51038, Enzo Life Sciences Inc.); UNC45A (catalog sc-101493, 1:50, Santa Cruz Biotechnology Inc.); villin (catalog sc-58897, 1:50, Santa Cruz Biotechnology Inc.); DRA (catalog ab83545, 1:100, Abcam); Rab 11 (catalog 71-5300, 1:25, Invitrogen); NHE3 (catalog NBP-82574, 1:500, Novus Biologicals); MYO5B (catalog HPA069773, 1:400, Sigma-Aldrich); Epcam (catalog AF960, 1:100, R&D); CFTR (catalog ab59394, 1:100, abcam); NA/K-ATPase (catalog MA5-32184, 1:100, Thermo Fisher); integrin-α2 (catalog ab181548, 1:100, Abcam); and E-cadherin (catalog 13-700445, 1:100, Thermo Fisher). For STED, F-actin (phalloidin, phalloidin-ATTO 550, Invitrogen) was used. For Western blot, the following antibodies were used: actin (catalog 5125, 1:1000, Cell Signaling); FLAG (catalog F3165, 1:1000, Sigma-Aldrich); GAPDH (catalog 14C10, 1:1000, Cell Signaling Technology); HSP90 (catalog 4874, 1:1000, Cell Signaling Technology); UNC45A (catalog 171328, 1:500, Abcam); and MYO5B (catalog PA5-49519, 1:500, Thermo Fisher).

### Intestinal organoid generation.

Intestinal organoids were generated from iPSC lines derived from 2 healthy donors and 2 UNC45A-deficient patients (P1 and P4). All lines were cultured in mTeSR 1 (Stem Cell Technologies) and passaged from a 60 mm petri dish to a 24-well plate coated with 25% hESC Matrigel (356231, Corning Inc.). For generation of UNC45A-deficient iPSC lines, UNC45A targeting sg1 was cloned in the vector pSpCas9(BB)-2A-GFP (PX458) (no. 48138, Addgene) (32). Transfection of control iPSCs was performed by cuvette electroporation using an Amaxa Cell Line Nucleofector electroporator (Lonza). Three to five days after electroporation, single transfected cells were FAC sorted (FACSAria, BD) based on expression of GFP and seeded in mTeSR1 (Stem Cell Technologies) for subsequent clonal line generation. Several clones were screened for UNC45A expression by Western blot (Supplemental Figure 5). In order to obtain intestinal organoids, iPSC colonies were dissociated into clumps using Gentle Cell Dissociation Reagent (07174, Stem Cell Technologies) and replated at a density of 6 × 10^3^ clumps per well of a Matrigel-coated 24-well tissue culture plate in mTeSR1. Cells were then differentiated into definitive endoderm by culturing the cells for 3 days in Definitive Endoderm (DE) Medium (STEMdiff Endoderm Basal Medium [no. 05111] + STEMdiff Definitive Endoderm Supplement CJ [no. 05113], Stem Cell Technologies). The cells were then cultured in mid- and hindgut (MH) medium (STEMdiff Endoderm Basal Medium [no. 05111] + STEMdiff Gastrointestinal Supplement PK [no. 05141] + STEMdiff Gastrointestinal Supplement UB (no.05142], Stem Cell Technologies) and free-floating mid- and hindgut spheroids appeared after 5 to 9 days of differentiation. Spheroids were then embedded in Matrigel domes and cultured in STEMdiff Intestinal Organoid Growth Medium (OGM) (STEMdiff Intestinal Organoid Basal Medium [no. 05111] + STEMdiff Intestinal Organoid Supplement [no. 05144] + l-Glutamine, Stem Cell Technologies), allowing the differentiation of organoids in 20 days. Organoid cultures were then passaged every 7 to 10 days, depending on density, size, and morphology. For organoid forskolin swelling assay, differentiated 3D organoids were stimulated with 10 μM forskolin (Tocris) for 90 minutes and imaged using MC120HD (Leica Microsystems). The organoids’ swelling was analyzed and percentage changes in perimeter were quantified using ImageJ. Only lumen-forming organoids were analyzed. For monolayer culture, one ibidi plate (8 wells) was used to generate a full 24-well Transwell plate (0.4 mm polyester membrane, 3470, Corning Costar). 3D organoids in Matrigel were collected and resuspended in 1 ml of Gentle Dissociation Reagent (Stem Cell Technologies) to depolymerize Matrigel and incubated 10 minutes at RT with gentle shaking. After washing in ice-cold DMEM, organoids were resuspended in 1 mL of TrypLE Express Enzyme (no. 12605010), pipetted up and down thoroughly, and incubated at 37°C for 10 minutes until a single-cell suspension was obtained. After adding 10 ml of ice-cold DMEM, cells were centrifuged at 200*g* and resuspended in Monolayer Growth Medium (IntestiCult OGMH Component A + IntestiCult OGMH Component B + Y-27632, Stem Cell Technologies), and 100 μL of the cell suspension was added to the top of each 24-well Transwell. The medium was replaced each 2 to 3 days for 21 days.

### Immunofluorescence and labeling of zebrafish sections.

WT Tupfel long-fin zebrafish strains were used and raised according to standard protocols. The *unc45A* mutant fish was a gift of Beth L. Roman (University of Pittsburgh, Pittsburgh, Pennsylvania, USA). A nonsense mutation T1964A creates a premature stop codon within the UCS domain (allele tr12, ref. 25).

For immunofluorescence, zebrafish larvae were fixed for 2 hours at RT in 4% PFA and incubated in 30% sucrose/0.1% PBST (0.5% Tween, Bio-Rad) overnight at 4°C. They were then frozen in Tissue-Tek OCT (Sakura) at –80°C and sectioned using a Cryostat (Leica Microsystems). Sections were incubated in blocking buffer (10% serum in PBST) and with primary antibodies overnight at 4°C. After washes with PBST, they were incubated with Alexa Fluor secondary fluorescent antibodies (Molecular Probes, Invitrogen), phalloidin–Alexa Fluor 568 (Molecular Probes), and DAPI. Sections were imaged on a LSM780 confocal microscope (Zeiss).

### TEM.

TEM was performed on human duodenal tissue and organoids as previously reported (17). PAS-cytochemistry at the EM level (33) was performed as previously described (20). Morphometry of catabolic organelles (lysosomes, autophagosomes: pooled) was performed on digital images using iTEM software (EMSIS). About 100 organelles per condition were measured (from at least 25 cells each, from 2 different samples). For zebrafish studies, 5 dpf larvae were collected and stored at 4°C in Trump’s fixative. Enhanced chemical fixation was performed in a mix of 4% paraformaldehyde and 2.5% glutaraldehyde in 0.1 mol/L cacodylate buffer overnight at 4°C. A 1.5-hour incubation in 1% OsO4 was followed by a 1.5-hour incubation with 2% uranyl acetate at ambient temperature. Larvae were then dehydrated through graded ethanol solutions, cleared in acetone, infiltrated, and embedded in Epon-Araldite mix (hard formula) (Electron Microscopy Sciences). Adhesive frames (11560294, Gene Frame, 65 μL; Thermo Fisher Scientific) were used for flat embedding, as previously described (34). Ultrathin sections were cut on an ultramicrotome (EM UC7; Leica Microsystems) and collected on formvar-coated slot grids (FCF2010-CU, EMS). Each larva was sectioned transversally in 5 different places in intestinal bulb with 20 μm or more between grids for examining the sample over a large region. Each grid contained at least 4 to 6 consecutive sections of 70 nm. TEM grids were observed using a JEM-1400 TEM (JEOL) operated at 120 kV, equipped with a Gatan Orius SC1000 camera (Gatan), and piloted by the DigitalMicrograph 3.5 program (Gatan). Microvilli length and density were quantified using Fiji on TEM pictures of at least 50 microvilli from 25 enterocytes of 3 larvae per condition.

### Human UNC45A protein structure modeling.

The 3D model of human UNC45a protein was constructed by homology modeling using Modeller software available on the @TOME server (35, 36). The structures of *D*. *melanogaster* (PDB entry: 3NOW) and *C*. *elegans* (PDB entry: 6QDL) UNC45 proteins were used as templates (% of sequence identity between templates with human UNC45a is in average of 34%). The stereochemical geometry of the models was assessed using the QMEAN scoring function (37). The best model (highest QMEAN score = 0.73) of human UNC45A was taken for further analysis of variants using the SDM protein stability analysis server (38) and UCSF Chimera (39).

### Statistics.

Statistical analysis was performed using GraphPad Prism 8. *P* ≤ 0.05 was considered significant. The tests used in each set of experiments are reported in the corresponding figure legend.

### Study approval.

P1 and P4 were recruited, with informed consent, at Necker Hospital as part of the Immunobiota Study approved by the Committee for Personal Protection Ile-de-France II. P2 was recruited in Oxford, United Kingdom, with informed consent under a protocol approved by the Oxford Gastrointestinal Illness Biobank. P3 was recruited in Dijon, France, with informed consent, under a protocol approved by the institutional review board of Université de Bourgogne Franche-Comté. P5 and P6 were recruited at the Training and Research Hospital, Ankara Child Health and Diseases, after informed consent for genetic testing and approval by local ethical committees. Control blood was obtained from healthy volunteers. Intestinal tissues from patients were obtained for diagnosis or therapeutic purposes. All procedures were performed in accordance with French and European Union animal welfare guidelines. All animal studies were approved by Sorbonne Université and Institut Curie (APAFIS#21323-2019062416186982 and APAFIS#6031-2016070822342309).

## Author contributions

RDL, CL, J Berthelet, FCH, ON, CRDC, SW, TV, AK, CR, J Baptista, MWH, BD, NL, CB, GFV, LF, AR, DF, ARJ, FDL, FDB, and GM performed experiments and/or interpreted data. MR, ICG, MMM, BD, NL, CB, and M Parisot provided critical technical or material support. CT, FL, EED, AMD, TM, FMR, and HHU provided clinical data and managed patients. M Parlato, RDL, and NCB drafted the manuscript with critical input from MWH, LAH, FRL, HHU, and GM. NCB, and M Parlato designed and supervised the project.

## Supplementary Material

Supplemental data

Supplemental table 1

Supplemental table 2

Supplemental table 3

## Version 1 05/16/2022

Electronic publication

## Version 2 03/23/2023

Author name corrected: Céline Revenu des Courtils
