# Supplementary material for: UNC45A deficiency causes microvillus inclusion disease–like phenotype by impairing myosin VB–dependent apical trafficking
Source: J Clin Invest. 2022 May 16;132(10):e154997. doi: 10.1172/JCI154997 (PMC9106349; doi:10.1172/JCI154997)
Supplement: Supplemental data [file jci-132-154997-s008.pdf]

Supplementary Figure 1

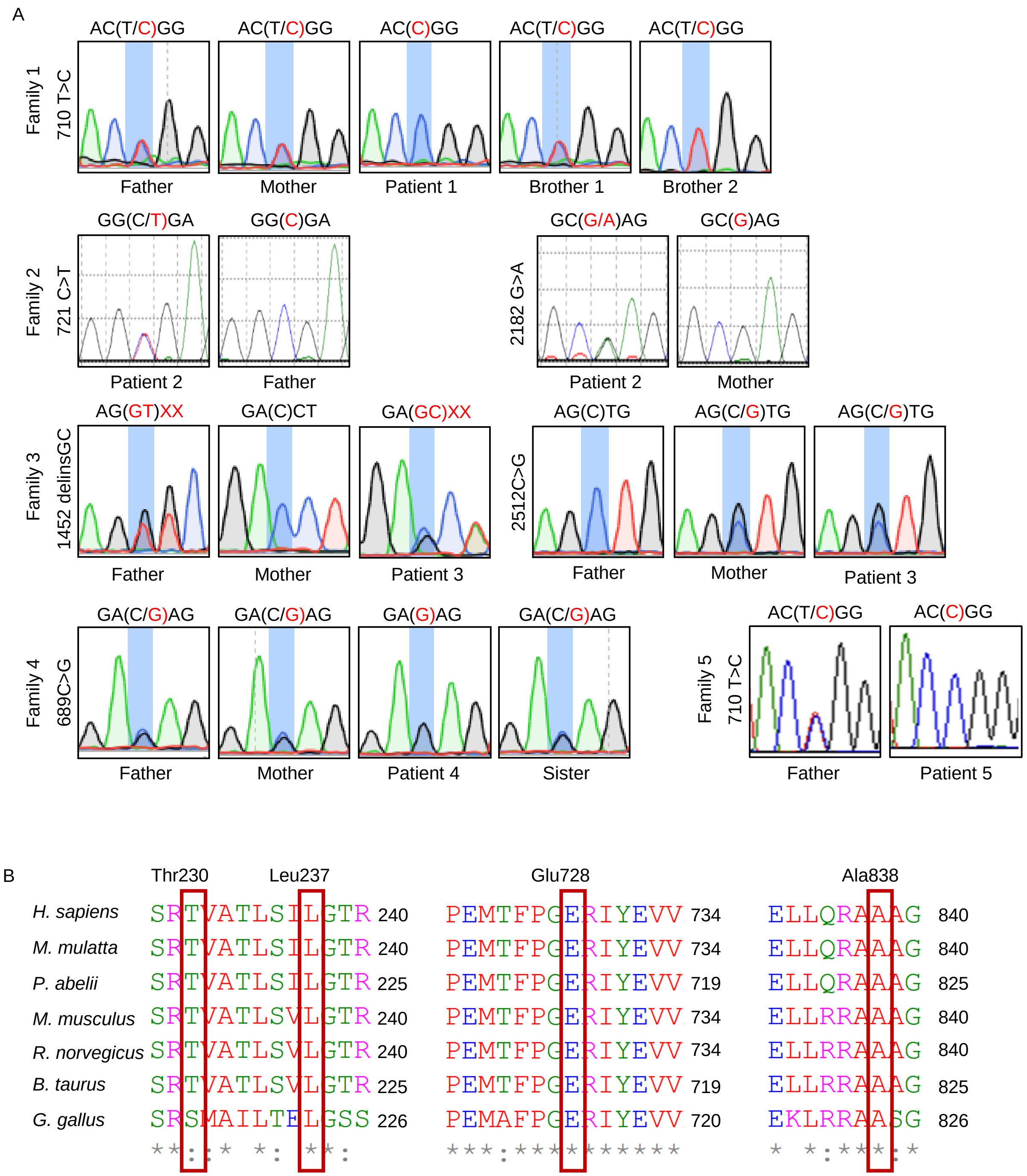

Supplementary Figure 1: A) Sanger sequencing showing segregation of the variants in each family. **B)** Alignment of UNC45A protein sequences showing conservation of the mutated amino acids (boxed) among species.

Supplementary Figure 2

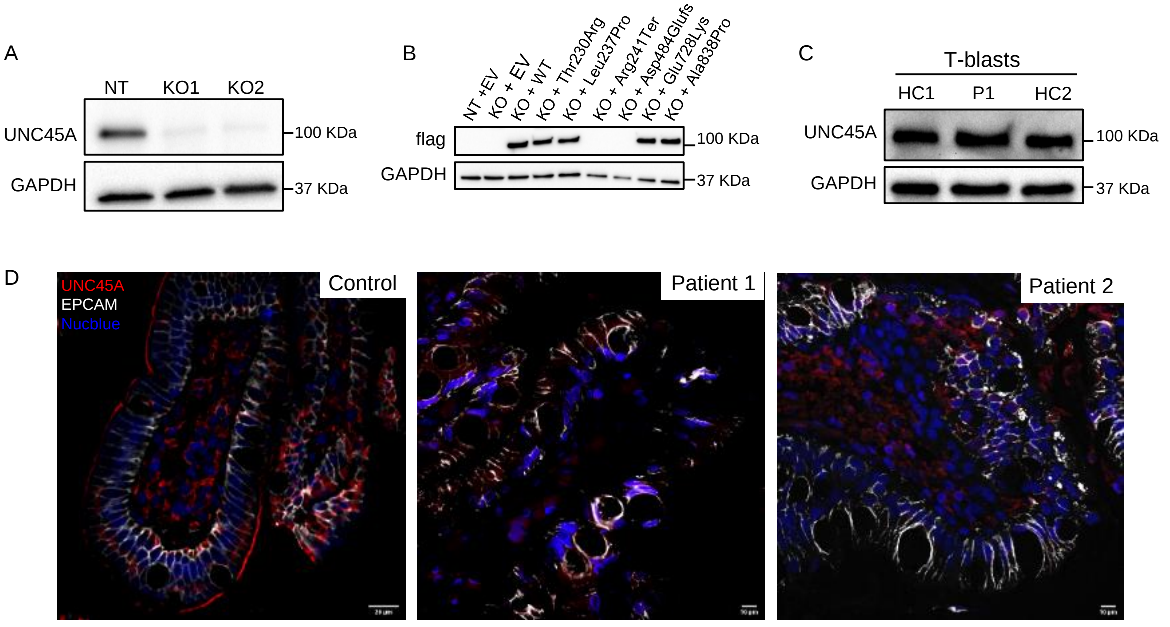

Supplementary Figure 2: A) UNC45A expression assessed by western blot (WB) in UNC45A^{KO} Caco-2 cells (CRISPR-guide 1 and CRISPR-guide 2) compared to non-targeting (NT) control cells. **B)** Flag expression assessed by WB in UNC45A^{KO} Caco-2 cells transduced with viral particles encoding empty plasmid (EV), flagged-WT or flagged- UNC45A mutants. GAPDH served as a loading control. **C)** UNC45A expression assessed by western blot in T-lympoblastoid cell lines derived from patient 1 (P1) and two healthy controls (HC). **D)** Confocal imaging showing localization/expression of UNC45A in duodenum of control (scale bar= 20 μm) and patients (scale bar = 10 μm).

Supplementary Figure 3

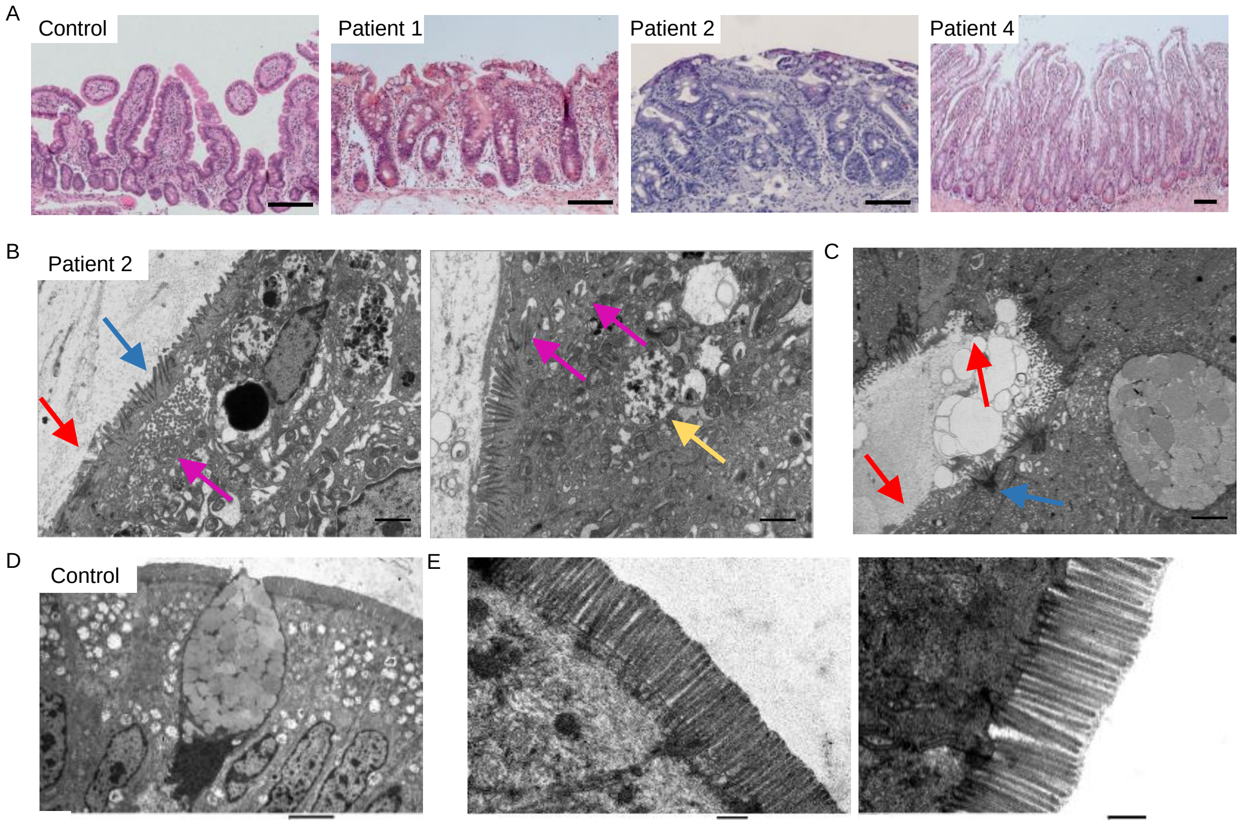

Supplementary Figure 3: **A)** H&E staining in control, P1, P2 and P4 duodenal biopsy scale bar = 100 μm. **B-C)**Transmission electron micrographs of Patient 2's duodenal enterocytes showing ultrastructural features of MVID: partially degraded microvilli inclusions (yellow arrowhead), defective microvilli anchored deep into the cytoplasm (blue arrowhead) or partially depleted (red arrowhead), basolateral microvilli (pink arrowhead) B, scale bar = 0.5 μm; C, scale bar = 2 μm; D-E) Transmission electron micrographs of control enterocytes, scale bar = 6 μm (D) and 1 μm (E) .

Supplementary Figure 4

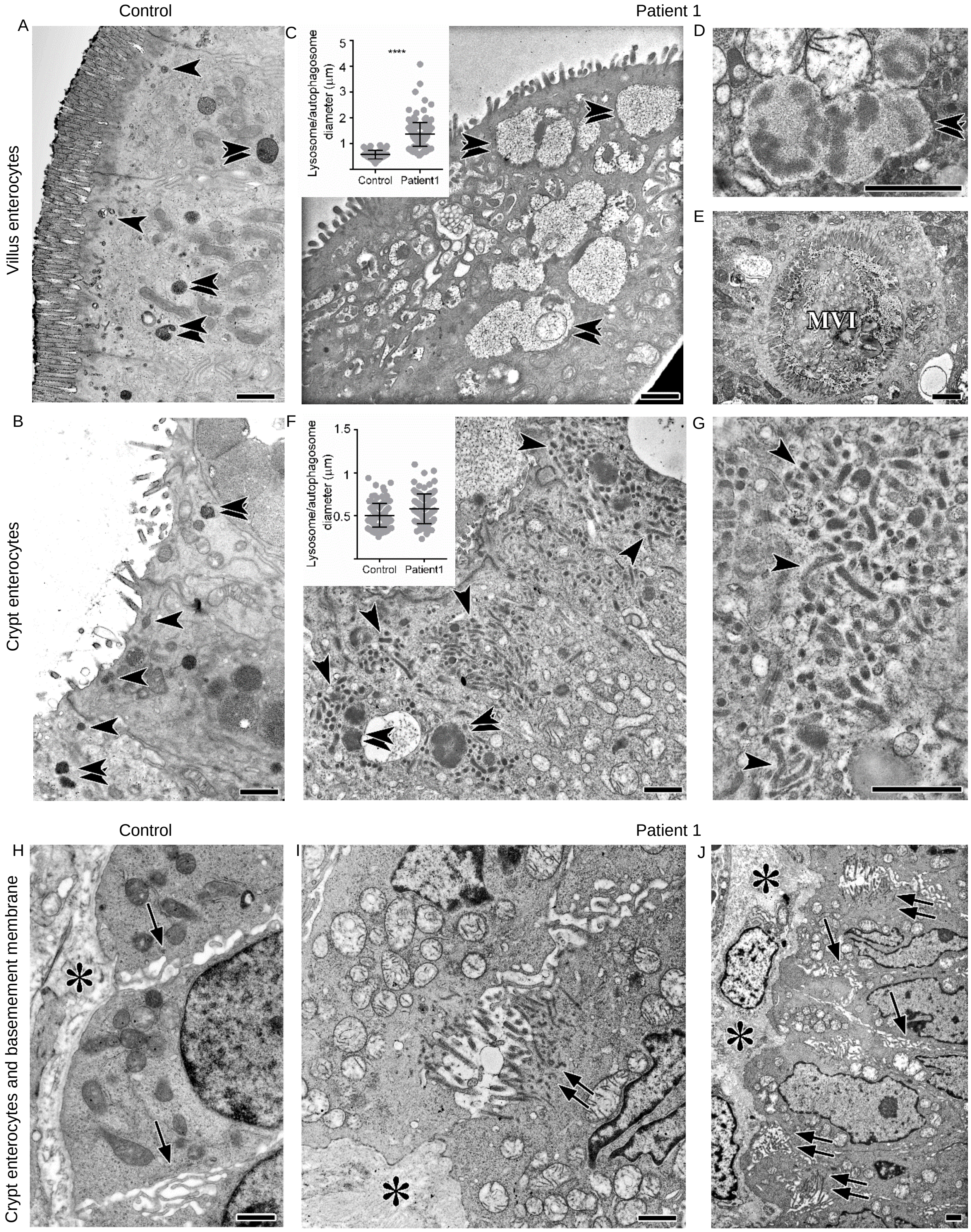

Supplementary Figure 4: PAS cytochemistry for EM on duodenum of control and Patient 1. **A-B)** Control enterocytes with normal apical microvilli (A,B), few normal sized PAS positive, dark vesicles (arrow-heads) and normal late endosomes/lysosomes (double arrow-heads in A-B). **C-G)** MVID features in Patient 1: i) scarce, short apical microvilli (C), ii) large lysosomes/autophagosomes (C,D), iii) classical microvillus inclusion (MVI) with ectopic brush border lining and terminal web (E), iv) subapical clusters of PAS positive vesicles and tubules (F, G). **H)** Control enterocytes with bent, finger-like cell protrusions (arrows) close to the basement membrane (*). I-J) Enterocytes from Patient 1 display irregularly arranged, straight microvilli (double arrows) with clear actin core, rootlets and adjacent ectopic terminal web close to the basement membrane (normal basolateral zones marked by single arrows), scale bars= 1µm.

Supplementary Figure 5

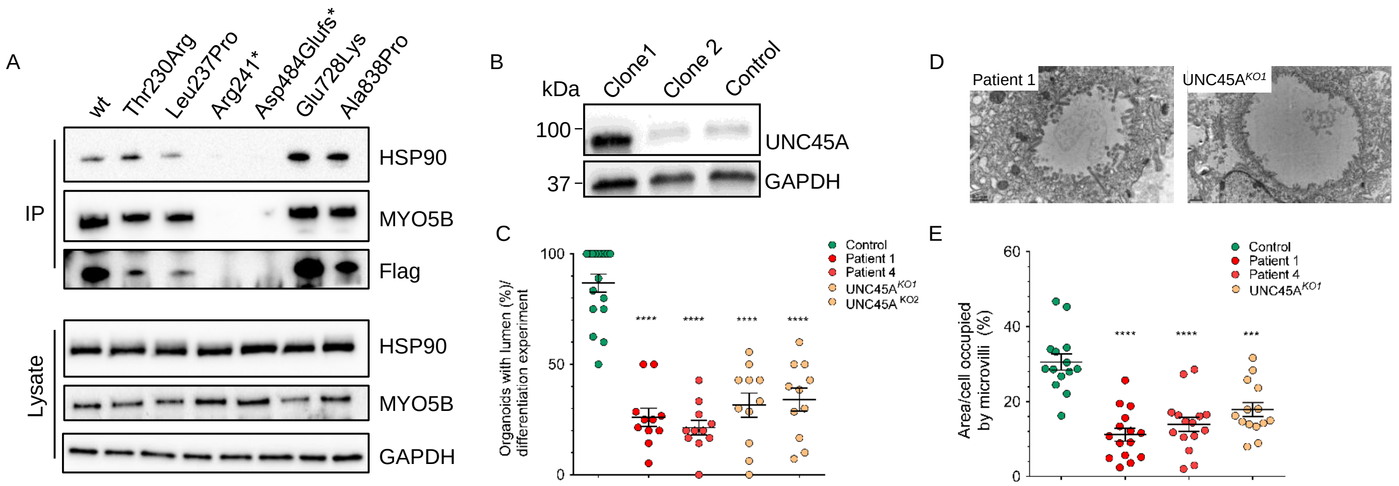

Supplementary Figure 5: A) HSP90, MYO5B and flag detection in UNC45A^{KO} cells complemented or not with myc/flag-tagged WT or myc/flag-tagged mutant alleles cell lysates immunoprecipitated with an anti-myc antibody (n=3). **B)** Western blot analysis of UNC45A expression in UNC45A^{KO} IPS cells generated with guide 1 (sg1) compared to control cells. GAPDH, loading control. **C)** Percentage of organoids forming central lumen, average +- SEM is shown. Two tailed Mann-Whitney test, **** p< 0.0001. **D)** TEM revealing microvillus inclusions in 3D organoids derived from patient 1 and in UNC45A^{KO1} organoids. (scale bar = 0.5 μm). **E)** Percentage of area/cell occupied by microvilli, average +- SEM is shown. Two tailed Mann-Whitney test, **** p< 0.0001; *** p=0.0002.

Supplementary Figure 6

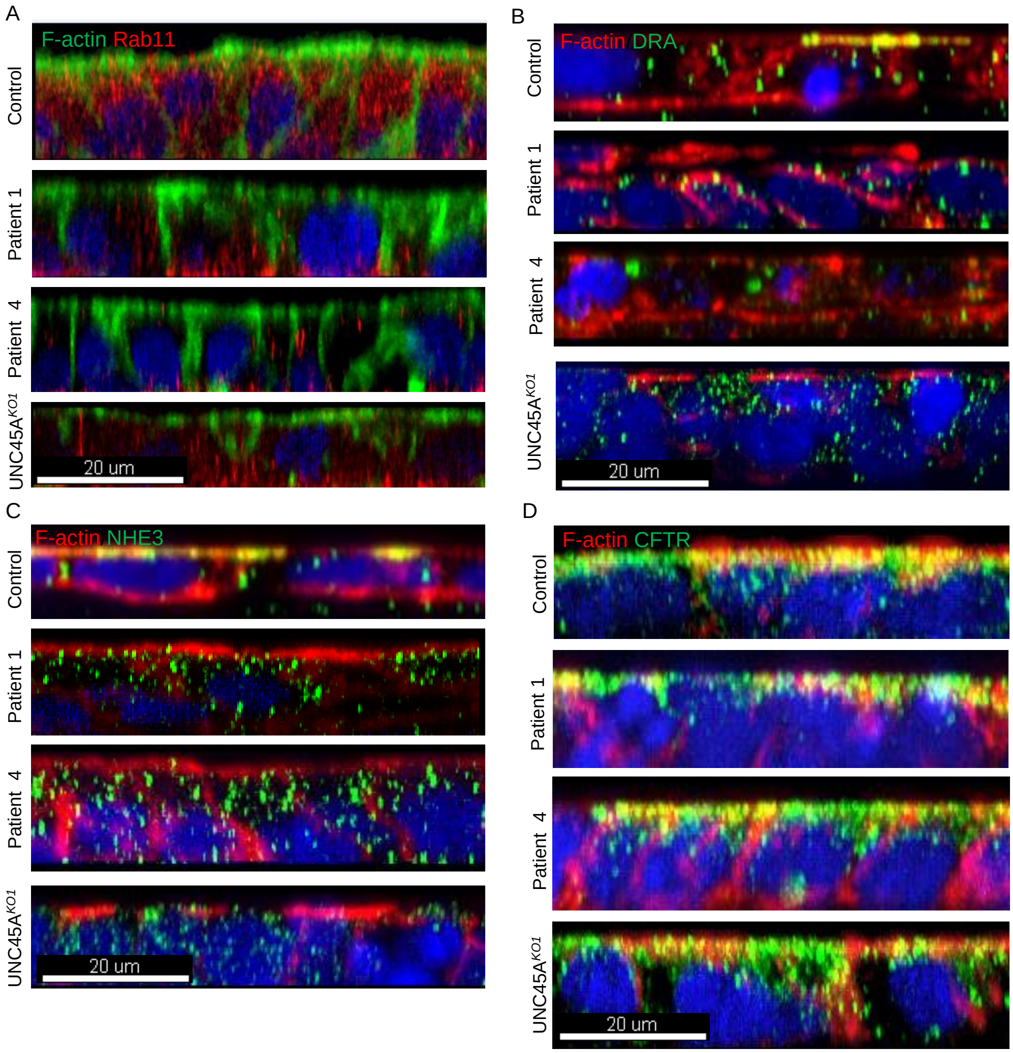

Supplementary Figure 6: Confocal imaging showing subapical and apical localization of Rab11 **(A)** and apical transporters, DRA **(B)**, NHE3 **(C)** and CFTR **(D)**, in many control enterocytes. Enterocytes monolayers derived by P1, P4 and UNC45A^{KO1} organoids appeared disorganized with dispersed RAB11 (A) and NHE3/DRA expression **(B-C)** but preserved apical CFTR localization **(D)**, scale bar = 20 μm, n=3.

Supplementary Figure 7

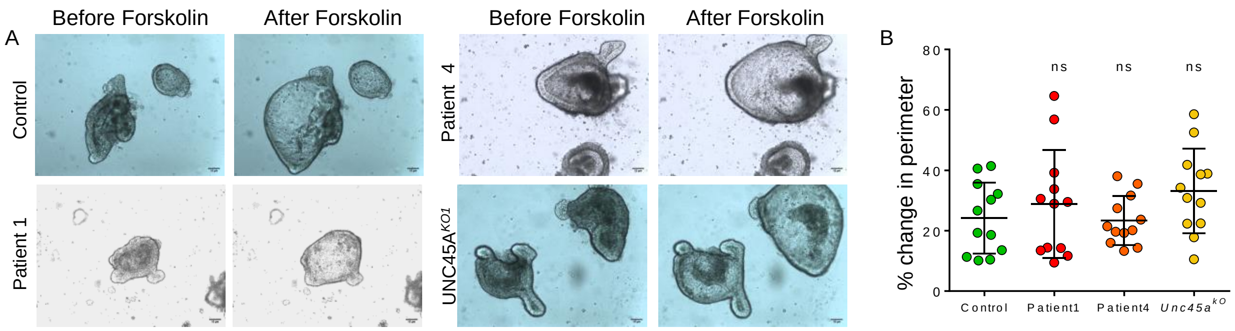

Supplementary Figure 7: A) Brightfield imaging of differentiated control, P1, P4 and UNC45A^{KO1} organoids before and 90 minutes after forsfokolin administration. **B)** Forskolin swelling as percent change in perimeter after 90 minutes forsfokolin administration. Only organoids forming lumen were taken into account. Ns: non significant, Two tailed Mann-Whitney test.

Supplementary Figure 8

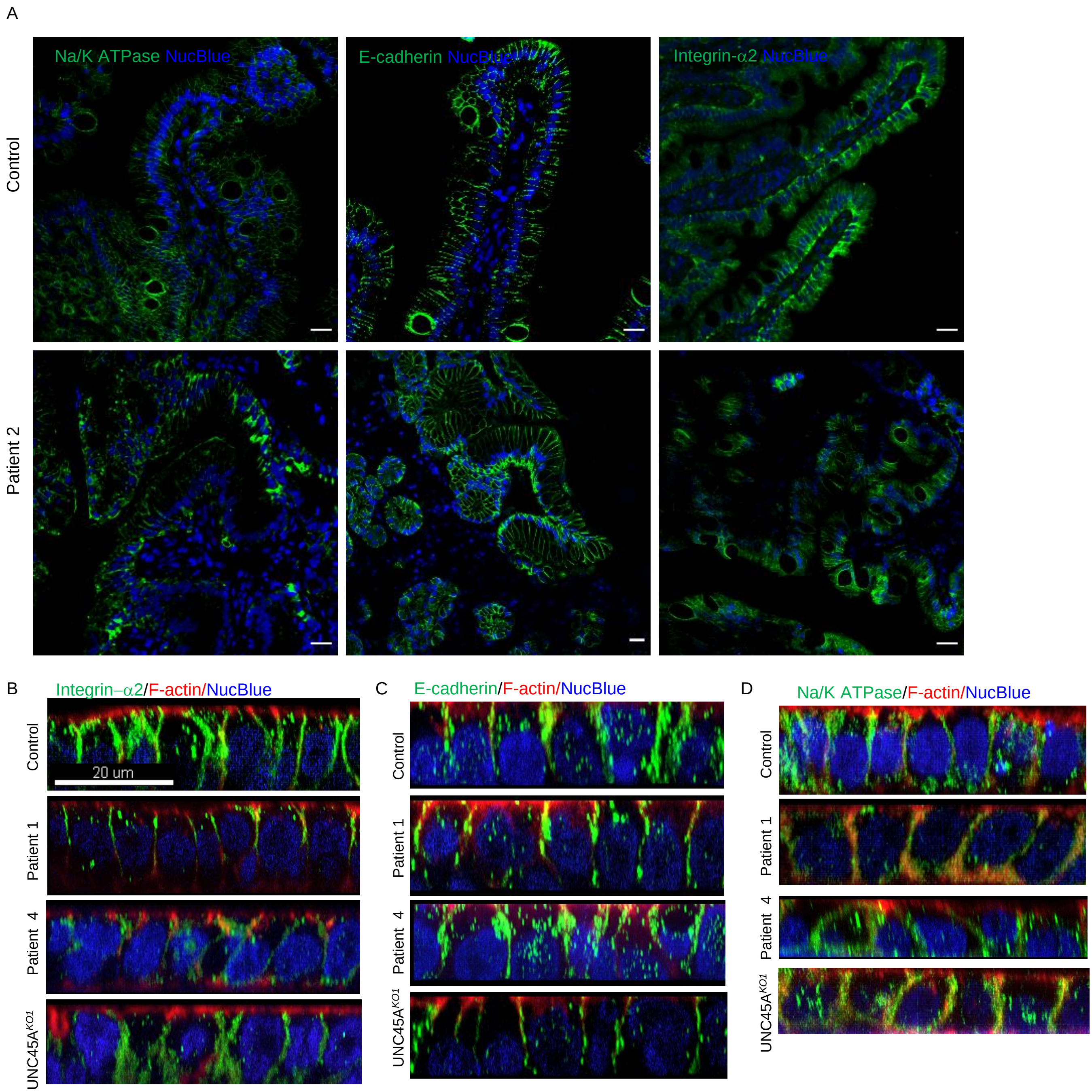

Supplementary Figure 8: A) Confocal imaging showing localization of Na/K ATPase, E-cadherin and integrin-α2 in duodenum of control and Patient 2, scale bar = 20 μm. **B-D)** Confocal imaging showing conserved lateral and basolateral localization of integrin-α2 (B) and E-cadherin (C) and Na/K ATPase (D), respectively, in enterocytes monolayers derived from P1, P4 and UNC45A^{KO1}, scale bar = 20 μm, n=3.

Supplementary Figure 9

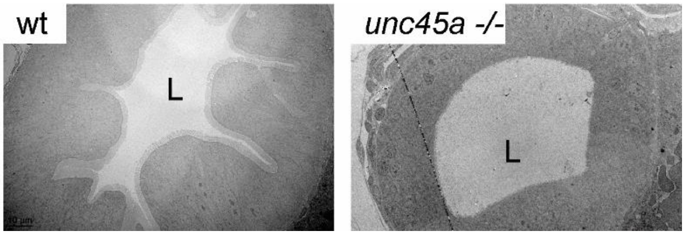

Supplementary Figure 9: Electron micrographs of thin sections of intestinal bulb of 5dpf wt and unc45a-/- mutant larvae showing lack of epithelial folds, scale bar = 10 µm. L: Lumen.
