# Supplementary material for: UNC45A deficiency causes microvillus inclusion disease–like phenotype by impairing myosin VB–dependent apical trafficking
Source: J Clin Invest. 2022 May 16;132(10):e154997. doi: 10.1172/JCI154997 (PMC9106349; doi:10.1172/JCI154997)
Supplement: Supplemental table 1 [file jci-132-154997-s009.pdf]

Table 1. Patient Characteristics

	P1	P2	P3	P4	P5	P6
**Origin**	Turkish	United Kingdom	French	French (West Indies)	Turkish	Turkish
**Current age**	6 y	2 y	22 y	10.5 y	died 93rd day	3 m
**Onset age**	3 weeks	1 week	1 week	4 days	1 day	5 days
**Severe diarrhea**	yes, with hypovolemic shock	yes	yes, intermittent until 10 y	yes	yes	yes
**Duodenal villous atrophy**	yes	yes	yes	yes	ND	ND
**Sub apical PAS accumulation**	yes	yes	yes	yes	ND	ND
**Sub apical CD10 accumulation**	yes	yes	ND	yes	ND	ND
**Electron microscopy**	Vesicles or tubulovesicular structures<br>MVI<br>Basolateral microvilli<br>large lysosomes/autophagosomes<br>Defective microvilli anchored deep in the cytoplasm<br>thickened terminal web region	Vesicles or tubulovesicular structures<br>Defective microvilli<br>Basolateral microvilli<br>large lysosomes/autophagosomes	ND	ND	ND	ND
**Treatment**	TPN<br>Total enterectomy	TPN	Exclusive enteral nutrition	TPN<br>Small bowel transplantation	TPN	TPN
**Cholestasis**	yes	no	yes	no	yes	yes
**Bone fragility**	Fracture of the left inferior part of the femur at 7th months and at 2 y<br>Fracture of the right wrist at 1 y	no	Fracture of the scapula at 7 y<br>Other fracture at 10 y	no	ND	One bone fracture upon delivery
**Deafness**	no	no	yes	no	no	ND

**Other manifestations**	no	no	blind: right anophtalmia, Peters anomaly & left pyramidal cataract / severe ID & behavioral disorder	ID	ND	ND

ND: not determined; ID: intellectual disability; TPN: total parenteral nutrition; MVI, microvillus inclusion.
