# Supplementary material for: UNC45A deficiency causes microvillus inclusion disease–like phenotype by impairing myosin VB–dependent apical trafficking
Source: J Clin Invest. 2022 May 16;132(10):e154997. doi: 10.1172/JCI154997 (PMC9106349; doi:10.1172/JCI154997)
Supplement: Supplemental table 2 [file jci-132-154997-s010.pdf]

Supplementary Table 2: Characteristics of UNC45A variants

	**Variant** NM_018671.4	**Consequence**	**SIFT**	**POLYPHEN**	**Mutation taster**	**CADD**	**gnomAD Allele frequence**
**P1 P5 P6**	c.710T>C	Leu237Pro	Deleterious	Possibly damaging	Disease causing	27.2	3.99e-6
**P2**	c.721C>T	Arg241*	Not scored	Not scored	Not scored	23	Not observed
	c.2182G>A	Glu728Lys	Deleterious	Probably damaging	Disease causing	27.4	6.39e-6
**P3**	c.1452delinsGCA	Asp484Glufs*17	Not scored	Not scored	Not scored	23	Not observed
	c.2512G>C	Ala838Pro	Tolerated	Probably damaging	Disease causing	25.5	Not observed
**P4**	c.689C>G	Thr230Arg	Deleterious	Possibly damaging	Disease causing	25	Not observed

CADD: Combined Annotation Dependent Depletion.
